# Greater power and computational efficiency for kernel-based association testing of sets of genetic variants

**DOI:** 10.1093/bioinformatics/btu504

**Published:** 2014-07-29

**Authors:** Christoph Lippert, Jing Xiang, Danilo Horta, Christian Widmer, Carl Kadie, David Heckerman, Jennifer Listgarten

**Affiliations:** ^1^eScience Research Group, Microsoft Research, Los Angeles, CA, 90024 and ^2^eScience Research Group, Microsoft Research, Redmond, WA, 98052, USA

## Abstract

**Motivation:** Set-based variance component tests have been identified as a way to increase power in association studies by aggregating weak individual effects. However, the choice of test statistic has been largely ignored even though it may play an important role in obtaining optimal power. We compared a standard statistical test—a score test—with a recently developed likelihood ratio (LR) test. Further, when correction for hidden structure is needed, or gene–gene interactions are sought, state-of-the art algorithms for both the score and LR tests can be computationally impractical. Thus we develop new computationally efficient methods.

**Results:** After reviewing theoretical differences in performance between the score and LR tests, we find empirically on real data that the LR test generally has more power. In particular, on 15 of 17 real datasets, the LR test yielded at least as many associations as the score test—up to 23 more associations—whereas the score test yielded at most one more association than the LR test in the two remaining datasets. On synthetic data, we find that the LR test yielded up to 12% more associations, consistent with our results on real data, but also observe a regime of extremely small signal where the score test yielded up to 25% more associations than the LR test, consistent with theory. Finally, our computational speedups now enable (i) efficient LR testing when the background kernel is full rank, and (ii) efficient score testing when the background kernel changes with each test, as for gene–gene interaction tests. The latter yielded a factor of 2000 speedup on a cohort of size 13 500.

**Availability:** Software available at http://research.microsoft.com/en-us/um/redmond/projects/MSCompBio/Fastlmm/.

**Contact:** heckerma@microsoft.com

**Supplementary information:** Supplementary data are available at *Bioinformatics* online.

## 1 INTRODUCTION

With next-generation sequencing data from larger and larger cohorts now being collected, the possibility of detecting even weaker genetic associations with disease is increasing. Such weak signal could provide invaluable insights into biological and disease mechanisms, as well as yield biomarkers for diagnosis and personalized treatment. However, even with large datasets becoming available, studies to detect important genetic signal remain underpowered, especially those rare variants—the most underpowered type of association whose signal lies in tests.

One approach to help alleviate this power problem is to group together genetic markers and then to test them jointly in a single test. Such an approach helps increase power in two ways. First, it can reduce the number of tests performed and hence the multiple testing penalty incurred. Second, the test aggregates weak signal within a set, and can also tag unmarked variants. Although a variety of competing methods for set tests have been proposed (Bhatia *et al.*, 2010; Han and Pan, 2010; Ionita-Laza *et al.*, 2011; Li and Leal, 2008; Liu and Leal, 2010; Madsen and Browning, 2009; Morgenthaler and Thilly, 2007; Neale *et al.*, 2011; Price *et al.*, 2010; Schwender *et al.*, 2011, Wu *et al.*, 2011; Zawistowski *et al.*, 2010), some of the most influential and widely used methods are those that use a sequence-based kernel in a variance component model (Chen *et al.*, 2013; Ionita-Laza *et al.*, 2013; Lee *et al.*, 2012a; Listgarten *et al.*, 2013; Liu *et al.*, 2007, 2008; Oualkacha *et al.*, 2013; Schifano *et al.*, 2012; Wu *et al.*, 2011). Improving power in these kernel-based models is the focus of this article. In particular, the main contribution of this article is improving power in two ways:

1. the statistical test used, showing that the non-standard likelihood ratio (LR) test in this setting can yield substantially more associations, and
2. several exact algebraic reformulations that yield dramatic improvements in runtime for certain classes of set tests, enabling far larger datasets to be analyzed. For example, on data from the Wellcome Trust Case Control Consortium (WTCCC), a gene–gene interaction score-test speedup achieved running time ∼2000 times faster than naïve computation of the test.

In the statistical genetics literature to date, practically no consideration has been given to the choice of statistical test for kernel-based set association tests. In particular, the choice of (frequentist) statistical test in this setting has uniformly been the score test (Ionita-Laza *et al.*, 2013; Lee *et al.*, 2012a; Liu *et al.*, 2007, 2008; Oualkacha *et al.*, 2013; Wu *et al.*, 2011), with the one exception being our recent work on how to conduct set tests in the presence of confounders, where an LR approach was used (Listgarten *et al.*, 2013). Band *et al.* (2013) also used an approximate Bayes Factor as a complement to the use of a score test. From a purely computational perspective, use of the score test would seem more convenient and efficient, as it requires parameter estimation only for the null model, whereas the LR test requires parameter estimation for both the null and the alternative model. In terms of power, various theoretical results claim the superiority of either the LR test or the score test, under different conditions. However, because these conditions are rarely met for real data, and because it is unclear how robust the theoretical results are to deviations from the required conditions, there is no clear theoretical guidance on which test to choose in practice. Therefore, here we conducted a systematic comparison between the two tests, using synthetic data, rare and common variants, with both case-control and continuous-valued phenotypes, and under various types of model misspecification. In so doing, we were able to assess the relative performance of the score and LR tests across a wide variety of settings when the ground truth was known. Finally, we applied the two tests to real data for 17 phenotypes to determine which of our synthetic settings were most likely applicable, finding that, overall, the LR test performed substantially better than the score test.

In addition to our systematic comparison of the score and LR tests in the standard setting, we also consider richer scenarios in which, for example, one may want to correct for confounding factors arising from family relatedness or population structure (Listgarten *et al.*, 2013), or testing for gene–gene interactions between variants from pairs of sets (e.g. genes) (Li and Cui, 2012). In such settings, there are two variance components—one consisting of a background kernel (e.g. to correct for confounding factors, or for main effects in a test for gene–gene interactions) and an additional component in the alternative model built from the set of interest. When the null model includes a background kernel, the time to run tests can be prohibitive. In particular, for the case where (i) the background kernel has full rank (as has been done traditionally when correcting for confounding factors), and (ii) where the background kernel is low rank but changing with each test (as in testing for gene–gene interactions), runtimes of state-of-the art LR and score tests can be dramatically decreased. Thus, we developed computational improvements and demonstrate their effectiveness through timing experiments.

## 2 RESULTS

### 2.1 Comparison of the score to the LR test

For genome-wide set tests based on a variance components model, the choice of statistical test has focused on the score test (Ionita-Laza *et al.*, 2013; Lee *et al.*, 2012a; Liu *et al.*, 2007, 2008; Oualkacha *et al.*, 2013; Wu *et al.*, 2011), with one exception being our recent work on how to conduct set tests in the presence of confounders in which an LR test was used (Listgarten *et al.*, 2013). As mentioned earlier, there appears to be no universally compelling reason to use one test over the other, as theoretical results are limited to specific situations that are not generally applicable. Next, we review some of the motivations and theorems that are often given for the use of one test over the other, as well as reasons that these arguments may not hold.

The usual reasons cited for use of the score test are that it is the locally most powerful test [e.g. (Chen *et al.*, 2013; Wu *et al.*, 2011)] and that it is fast to compute because the parameters of the alternative model need not be estimated—this computation is usually the most expensive one. However, rarely does there seem to be a discussion about why the ‘locally’ most powerful test is the desired one—locality here refers to hypotheses that are close/local to the null hypothesis. Sometimes, the argument is made that hypotheses further away from the null have such strong signal that any statistical test will find them. As we will show and explain, this is not an argument that the score test will have the most power in practice. On the other hand, for so-called simple hypotheses, the LR test is the uniformly most powerful test according to the Neyman–Pearson lemma. A simple hypothesis is one in which a null hypothesis (e.g. some parameter of interest, α = 0) is compared with a single alternative hypothesis (e.g. $\alpha =\alpha_{0}$). However, in most applications, including set tests in genetics (and those examined herein), the alternative hypothesis is a composite one—consisting of a range of viable parameter values (e.g. α > 0), so the Neyman–Pearson lemma does not apply.

One connection between the score and LR tests is that the score test can be interpreted as an approximation to the LR test in the neighborhood of the null hypothesis. In particular, if one fits a parabola to the log likelihood at the null hypothesis (a good approximation locally), then the resulting LR test statistic is equal to a score statistic, for a score statistic that uses the observed rather than the more traditional expected information (Buse, 2007). (Note that the observed information is asymptotically equivalent to the expected information, and therefore the LR test, in a local region, is asymptotically equivalent to the traditional score test (Buse, 2007), as well as the variant of the score test used in this paper.) However, because the LR test is not limited to a parabolic approximation of the likelihood surface, the LR test can, in principle, discover parameter values yielding larger likelihoods than those implied by the score test. If such a discovery happens for tests that are truly non-null (and happens less so for true nulls), then the LR test statistic will yield more power. If, however, this discovery happens for many truly null tests, creating relatively large LR test statistics under the null (effectively shifting the critical region towards larger test statistics), then the score test could yield more power. As a consequence, one can see intuitively why the score test may sometimes yield more power than the LR test and *vice-versa*.

In addition to these theoretical arguments for and against each test, there are further complications in that a variety of options are available for the score test. In practice, the so-called ‘score-based’ test is often used [e.g. SKAT (Wu *et al.*, 2011] rather than a more traditional version of the one-sided score test (Molenberghs and Verbeke, 2003). Theoretically, these two versions of the score test are asymptotically equivalent except that the traditional variance component score test uses a mixture of two distributions as an asymptotic null distribution (Molenberghs and Verbeke, 2003), whereas the score-based test can use a finite-sample null distribution, as used in SKAT. The latter null distribution can be derived analytically under the assumption that all nuisance parameters are known (see Supplemental Section 5.1 and (Davies, 1980; Goeman *et al.*, 2006)). In our experiments, we chose to use a score-based test as in SKAT because this is the standard in the genetics community (Chen *et al.*, 2013; Lee *et al.*, 2012a and 2012b; Oualkacha *et al.*, 2013; Wu *et al.*, 2011).

In summary, it is not clear whether one should use the score or the LR test to achieve maximal power. Therefore, we investigated this issue empirically, finding that on synthetic phenotype data, indeed, either test could outperform the other. For extremely small effect sizes, the score test offered greater power, while for larger effect sizes, the LR test offered better power. Analyzing 17 real datasets, we found that the LR test substantially outperformed the score test (Table 3). Next, we go in detail through these experiments. For simplicity, in the synthetic experiments, we focus on experiments that do not use a background kernel. However, when analyzing the real data, we analyze the datasets both with and without a background kernel for completeness.

### 2.2 Synthetic experiments

First, using real SNPs and synthetic phenotypes, we examined power and control of type I error across a variety of settings, including exclusively either common (based on WTCCC) or rare variants (based on BMI), and either Gaussian or binary phenotypes. For power experiments, the causal signal was precisely from all SNPs in the set being tested (except as noted in the section on model misspecification).

For binary phenotypes, theoretically, a generalized linear model (e.g. logistic) is a more appropriate choice than a linear model. However, exact inference for logistic models in this setting is generally intractable, requiring approximations (e.g. Le Cessie and van Houwelingen, 1995). One approximation that is commonly used for the logistic score (Wu *et al.*, 2011) is derived from the Laplace approximation to the quasi likelihood (Breslow and Clayton, 1993) . The utility of this approximation compared to use of a linear model, which is less appropriate but can be evaluated without approximations, has not, to our knowledge, been fully investigated. Therefore, we included in our experiments, both a linear and an approximate logistic score test, finding little, if any, difference between the two.

As shown in Tables 1 and 2, the type I error was controlled for all methods in all settings (i.e. no significant deviations from expectation were found). Each entry in the table is estimated from ∼1 million tests from the null distribution. We examined thresholds for which it remained practical to run experiments, which was as low as α=1×10^−5^.

**Table 1.** Type I error for common variants sets

Algorithm	$\alpha =10^{-5}$	$\alpha =10^{-4}$	$\alpha =10^{-3}$
Gaussian phenotype			
Linear score	1.3 × 10^−5^	1.1 × 10^−4^	1.0 × 10^−3^
Linear LR test	1.4 × 10^−5^	1.1 × 10^−4^	1.0 × 10^−3^
Binary phenotype			
Linear score	7.0 × 10^−6^	1.1 × 10^−4^	9.7 × 10^−4^
Linear LR test	1.0 × 10^−5^	1.1 × 10^−4^	1.0 × 10^−3^
Logistic score	7.0 × 10^−6^	1.1 × 10^−4^	9.7 × 10^−4^

No statistically significant deviations from expectation according to binomial test with significance level of 0.05.

**Table 2.** Type I error for rare variant sets

Algorithm	$\alpha =10^{-5}$	$\alpha =10^{-4}$	$\alpha =10^{-3}$
Gaussian phenotype			
Linear score	9.9 × 10^−6^	1.0 × 10^−4^	9.7 × 10^−4^
Linear LR test	6.9 × 10^−6^	1.1 × 10^−4^	1.0 × 10^−3^
Binary phenotype			
Linear score	1.4 × 10^−5^	9.6 × 10^−5^	9.7 × 10^−5^
Linear LR test	1.6 × 10^−5^	1.0 × 10^−4^	9.8 × 10^−4^
Logistic score	1.4 × 10^−5^	9.9 × 10^−5^	1.0 × 10^−3^

No statistically significant deviations from expectation according to binomial test with significance level of 0.05.

**Fig. 1.**
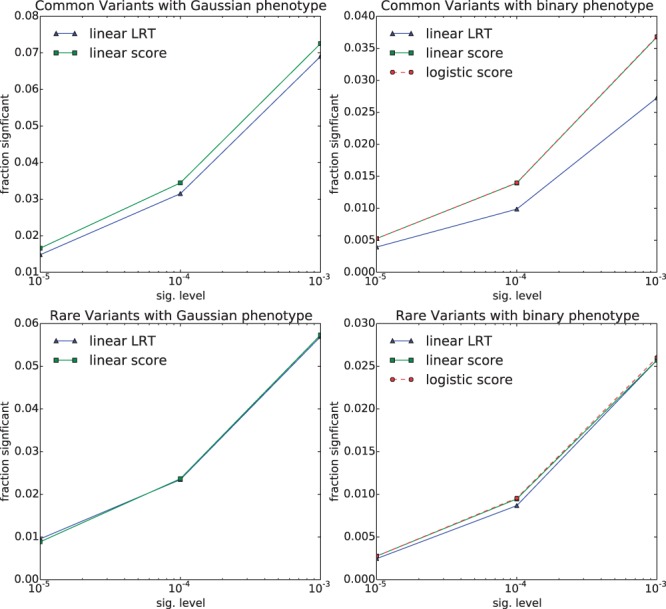
Power on synthetic data for each method in each setting, for the lowest signal strength, $h^{2}=0.001$. Fraction of tests deemed significant across various significance levels for each method is shown on the vertical axis. The threshold for significance is shown on the horizontal axis. Other signal strengths are shown in Figure 2 and Supplementary Figures S1 and S2

**Fig. 2.**
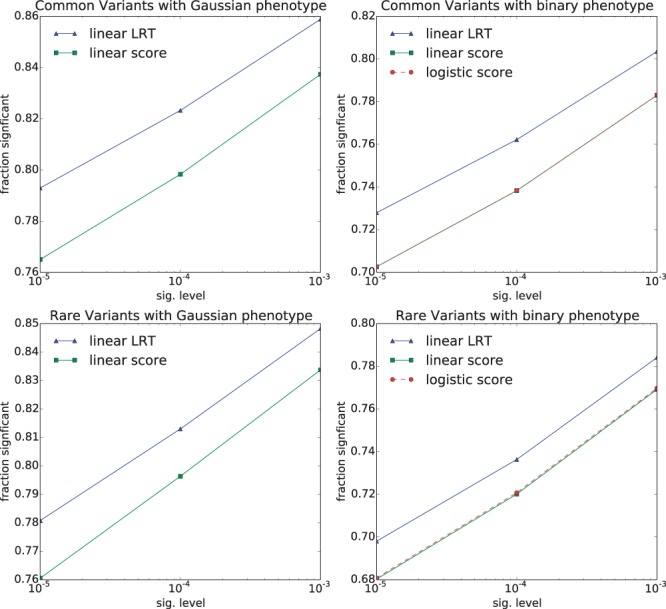
Power on synthetic data for each method in each setting, for signal strength, $h^{2}=0.01$. Fraction of tests deemed significant across various significance levels for each method is shown on the vertical axis. The threshold for significance is shown on the horizontal axis. Other signal strengths are shown in Figure 1 and Supplementary Figures S1 and S2

After establishing control of type I error, we then systematically investigated power, using four different levels of effect size for the causal SNPs (which were precisely those SNPs being tested), of $h^{2}=0.001,0.01,0.1,0.5$, and across a range of significance thresholds $10^{-5}$, $10^{-4}$ and $10^{-3}$ (the same thresholds used for the type I error experiments). We found that for the lowest signal strength ($h^{2}=0.001$), the score test yielded slightly more power than the LR test, consistent with the notion that the score test is locally optimal (Fig. 1). For the other signal strengths ($h^{2}=0.01,0.1,0.5$), we found that the LR test yielded more power than the score test at each level (Fig. 2, Supplementary Figs S1 and S2), and in aggregate (Supplementary Fig. S3). The setting with the largest gain for the score test ($h^{2}=0.001,\alpha =10^{-3},$ common variants, binary phenotype) showed a 25% relative gain in power for the score test over the LR test. However, this setting has so little signal that even for the score test, power was only 4%. The setting with the largest gain for the LR test ($h^{2}=0.01,\alpha =10^{-5},$ common variants, Gaussian phenotype) showed a 12% relative gain in power for the LR over the score test, consistent with our real-data experiments.

It is interesting to note that in all settings, the logistic score test performed nearly identically to the linear score test. Thus, although use of a score test allows one to use an approximate logistic model, our results suggest that in practice this logistic model approach confers little benefit over the linear model. It is possible that an approximate logistic LR test may confer some advantage, but we have not yet examined this possibility.

In all of our experiments, we used a prevalence of 50%, and strong deviations from this prevalence could change the results. In our real data experiments (see below, and Section 4), we found that $h^{2}$ was typically on the order of a few percent (in the regime where, on synthetic data, the LR test tends to outperform the score test).

### 2.3 Real data

After establishing type I error control and regimes of superior power for each of the score and LR tests, we next applied these two tests to several real datasets: all seven phenotypes from the WTCCC, and eight phenotypes from the Atherosclerosis Risk in Communities (ARIC) dataset, counting the number of associations found to be significant at the Bonferroni threshold (Table 3, Supplementary Figs S6 and S7). We additionally analyzed a dataset for Warfarin dosing, and a rare variant body mass index (BMI) dataset, neither of which yielded many significant associations, and in both cases, yielded the same number for both the score and LR tests when using no background kernel: Warfarin yielded five associations, whereas BMI yielded none (Supplementary Fig. S8).

**Table 3.** Number of significant associations on real datasets

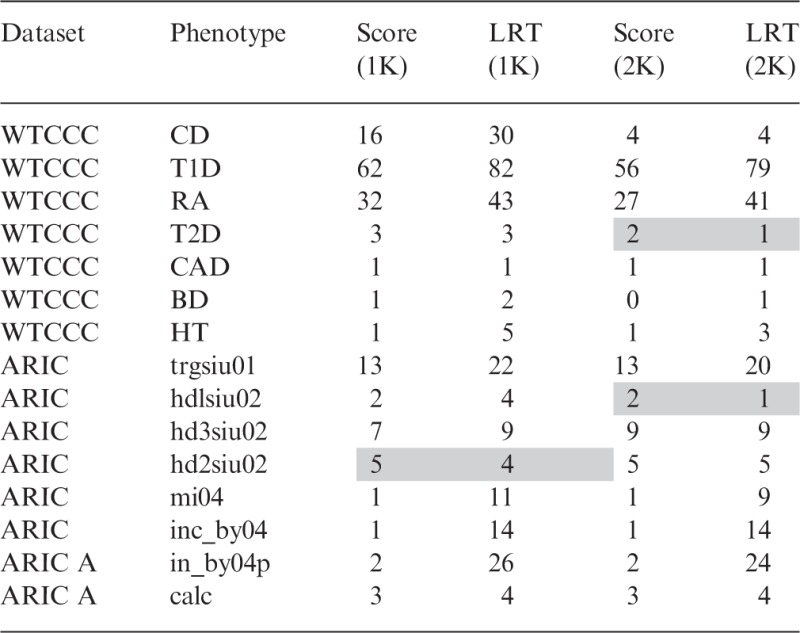

Number of significant associations on several real datasets using a Bonferroni-corrected threshold α = 0.05, when no background variance component is applied (1K), and when a background variance component computed from all SNPs is used to correct for potential confounding factors (2K). Grayed boxes denote cases where the score test found more associations than the LR test.

Although one can never be certain about what are true-positive results and true-negative results on real data, we conducted our experiments with the knowledge that in our simulations, the type 1 error was controlled, and therefore, reasonably assumed the same for these real datasets. This assumption, however, requires that the correct background kernel (perhaps none) be used. Thus, for these experiments, we performed a sensitivity analysis, applying our methods both with no background kernel, and with one (based on all SNPs). There were few differences between these two extremes (Table 3), indicating that on these data, there is little sensitivity to the choice of background kernel. This lack of sensitivity is unsurprising, as we originally chose these datasets with the intent of using only models with no background kernel for simplicity, and then added the background kernel analysis to verify our assumption that no kernel was needed.

The LR test identified as many or more significant sets than the score test on all but two of the real phenotypes (Table 3). Although one cannot make definitive conclusions from this limited number of datasets, our results suggest that the regime in which the score test outperforms the LR test is not commonly seen, and that in this regime, the difference in performance is minimal. This lies in contrast to the large differences sometimes seen when the LR test outperformed the score test.

As an added validation for analysis of the three WTCCC immune-related datasets (Crohn’s disease, T1 diabetes and Rheumatoid Arthritis), we downloaded known disease-associated loci from http://immunobase.org to see how many sets for each method were already known. For two of the datasets, the LRT test found more validated hits than the score test. For the third dataset, the number was the same for both tests (Supplementary Table S2).

One interesting point is that the increase in the number of associations when using the LR test over the score test on the real datasets (Table 3) is somewhat larger than that suggested by the increase in power observed on synthetic data (Figures 1 and 2). Thus, we conducted additional experiments under various forms of model misspecification to investigate whether we might identify the source of this difference.

### 2.4 Synthetic data experiments under various types of model misspecification

The first form of model misspecification we considered is related to the fact that, on real data, there is often a broad polygenic background signal. In such a setting, our variance component model becomes misspecified because the polygenic background would not be identically and independently (iid) Gaussian-distributed. To investigate how the presence of a polygenic background signal might influence the difference between our two statistical tests, we generated real-valued phenotypes from the WTCCC SNPs. Now, rather than adding iid Gaussian noise to the phenotype as in the previous experiments, we added a non-iid polygenic background signal. In particular, we used all of the even chromosome SNPs to generate effects drawn from a zero-mean Gaussian with variance 1.0, and tested only gene sets on the odd chromosomes. Then, we checked to see if the type 1 error was still controlled for each statistical test—it was (Supplementary Table S1). Next, for power experiments, we added in gene-specific signal (all SNPs within a gene, as before, and only from odd chromosomes) to this polygenic background, with gene SNP effects drawn from a zero-mean Gaussian with variance equal to 0.001, 0.01 and 0.1 (yielding, respectively, gene-specific foreground *h*^2^ = 0.001, 0.01, 0.1). In this setting, we observed similar trends to those in Figures 1 and 2 (Supplementary Fig. S5).

Having identified differences in performance because of polygenic background model misspecification, we next investigated how other forms of model misspecification might affect deviation between the two tests. First, we repeated the experiments from Figures 1 and 2 for $h^{2}=0.5$, but reducing the proportion of causal SNPs from all SNPs within a gene, down to 50%, 10% and 1% (always using at least one SNP). In this setting, we observed that the LR test yielded increasingly more power than the score test as the percentage of causal SNPs was decreased (Supplementary Fig. S4). The type 1 error was still controlled in this setting, as the null hypothesis was the same as in Table 1.

In both cases of model misspecification investigated, with increasing misspecification, the improvement of the LR test over the score test increased (Supplementary Fig. S4 and S5).

### 2.5 Computational speedups for score and LR tests

We now describe methods for improving the computational efficiency of both the LR and score tests. When testing one variance component in a one-component model, the asymptotic time complexity for the score and the LR tests are the same ($O\left(N^{3}\right)$ individuals when $k_{1}$ SNPs are being tested with $k_{1}\geq N$, and otherwise $O(Nk_{1}^{2})$). Additionally, the computation times are the same in practice (Table 4). However, in many settings, one needs to use a second variance component, which can change the absolute and relative computational cost of the two tests. Examples of settings with a two-variance components model include (i) a null model that contains a background kernel to correct for confounding factors (owing to, for example, family relatedness and population structure) (Listgarten *et al.*, 2013), and (ii) gene–gene interaction tests where the null model contains a background kernel for the additive gene terms (Li and Cui, 2012). There are two settings where computations are extremely expensive, and where we have developed new algorithms to improve their computational cost:

1. The first is when the background kernel is full rank, such as would occur when using a background kernel computed from all SNPs, when that number of SNPs is greater than the number of individuals. Here we have made the LR test dramatically more efficient, even while correcting for proximal contamination (Lippert *et al.*, 2011; Listgarten *et al.*, 2012).
2. The second case is when both the background kernel and the foreground (alternative model) kernel are jointly low rank. Here, we sped up the score test, dramatically so when the background kernel changes with each test.

**Table 4.** Runtimes and time complexity for the 13,500 WTCCC dataset

Algorithm	Time	Time complexity
One variance component model		
SKAT (Wu *et al.*, 2011)	0.03 s	$O\left(Nk_{1}^{2}\right)$
FaST-LMM-set score	0.03 s	$O\left(Nk_{1}^{2}\right)$
FaST-LMM-Set LR test	0.04 s	$O\left(Nk_{1}^{2}\right)$
Two variance component model full rank background kernel		
FaST-LMM-set score	2 s	$O\left(N^{2}k_{1}\right)$
FaST-LMM-set LR test	1.6 h	$O\left(N^{2}k_{1}\right)$
LMM-Set LR test (before improvement)	150 h	$O\left(N^{3}\right)$
Two variance component model low rank background kernel		
FaST-LMM-set score	See text	$O((N+k_{g})k_{1}^{2}$)
LMM-set score (before improvement)	See text	$O\left(N^{2}k_{1}\right)$
FaST-LMM-set LR test	See text	$O\left(N{\left(k_{g}+k_{1}\right)}^{2}\right)$

Runtimes on a single core and time complexities for various linear set tests, both without a background kernel (one variance component model) and with (two variance component model) after applying our improvements with exceptions noted. The time reported is the time per test averaged over 13 850 tests from the WTCC1 type 1 diabetes dataset. Runtimes and complexities for the two-variance full rank cases exclude the *O*(*N*^3^) computations shared across all tests and done upfront (2 s, when amortized over the 13 850 tests). The logistic score model had approximately the same timing as the linear score, and so here we report only the linear score. For the LR test, the time includes the 10 permutations that are required. Regarding the notation for time complexity, $k_{g}$ and $k_{1}$ refer to the size of the background and test components, respectively.

We now briefly give some intuition on the speedups and refer the reader to the Supplementary Methods Sections S2 and S5 for details.

#### Full rank background kernel speedups

For the case where precisely one of the two variance components is full rank (has more SNPs than individuals, as might happen when correcting for family-relatedness and population structure), we have developed a new approach for the LR test in which expensive computations (matrix inverses and determinants) are replaced by cheaper low-rank-update versions of them. For example, when $\text{Σ}=\sigma_{e}^{2}I+\sigma_{g}^{2}K_{g}+\sigma_{1}^{2}K_{1}$, where $K_{g}$ and $K_{1}$ are the background and test kernels, respectively (see Section 4), and the number of SNPs in $K_{1}$, $k_{1},$ is less than N, then, rather than taking the inverse of Σ (an N×N matrix)—a computation with time complexity $O\left(N^{3}\right)$—one can instead use the matrix inversion lemma (assuming that the inverse of $K_{\text{g}}$ is known, as it might be when re-using it for every SNP when correcting for population structure, for example) so that the time complexity becomes only $O\left(N^{2}k_{1}\right).$ This approach is particularly useful in settings where the background kernel remains constant across all or many or all tests (Table 4). However, even when this full-rank background kernel changes in a low rank manner with every test (as it would when correcting for proximal contamination), our improvement still allows the bottleneck computation to be performed only once per dataset. Furthermore, even when the background kernel changes entirely with every test (as in gene-gene interaction tests), our computations restrict the bottleneck computations to occur just once per test, rather than J times, where J is the number of optimization iterations. We also provide an efficient algorithm for the two kernel score test when the background kernel has full rank, although, others have developed similar methods for this case (Chen *et al.*, 2013; Oualkacha *et al.*, 2013), but these do not correct for proximal contamination.

#### Combined low rank variance component speedups

When the combined variance components are low rank in that the combined components have fewer SNPs than there are individuals, we previously showed how to make the LR test linear in N (Lippert *et al.*, 2011; Listgarten *et al.*, 2013). This algorithmic improvement can have a dramatic result on timing (see low rank versus full rank LR test under the ‘Two Variance Component’ header in Table 4), but, even with these computational improvements to the LR test, there are situations where computations are impractical—for example, when testing for gene–gene interactions (Li and Cui, 2012) with large *N*. Consequently, we have in addition developed a low rank algorithm for the score test. For large cohort sizes (e.g. the 13,500 individual WTCCC dataset used in the two-component timing experiments, which used all phenotypes as controls and included related individuals), the time (and memory) savings were substantial. For example, when testing a set of size 14 for gene–gene interactions using a low rank background kernel containing 150 SNPs, our low rank score test was ∼2000 times faster than the naïve implementation (1 s versus 32 min). Our efficient LR test in that same setting took 3.5 min.

It is worth noting that when using these low rank speedups, the time complexity for the score test remains the same between one-component and two-component models. However, this is not the case for the LR test, which requires an additional factor I—the number of optimization steps used to fit the alternative model. A typical number for *I* in our experience is around 20. Furthermore, because the LR approach requires on the order of 10 permutations (see Section 4), the LR test is expected to be around 200 times slower than the score test in this case (without specialized caching of expensive computations), and as we observed in practice (see previous paragraph). For the one-component model, caching permutations for the LR test is trivial, and is reflected in the timings in Table 4.

Previous work in speeding up the score test for two-component variance component models are somewhat limited—for example, requiring that the null model (and variance parameter) remain constant across all tests (Oualkacha *et al.*, 2013)—a condition not always met, such as in testing for gene–gene interaction (Li and Cui, 2012). These methods also cannot account for proximal contamination (Lippert *et al.*, 2011; Listgarten *et al.*, 2012) efficiently. Furthermore, these approaches cannot reduce the dependence on the number of individuals studied to a linear time; rather they can at best reduce it to a quadratic one (Chen *et al.*, 2013; Oualkacha *et al.*, 2013) even when the similarity matrix is low rank. Finally, their methods are quadratic in memory, whereas ours are linear in both time and memory in the low rank setting.

## 3 DISCUSSION

We have presented two strategies for increasing power in gene-set association studies. First, we investigated the difference in power between an LR and a score test, finding that, although on synthetic data, the score test outperformed the LR test when effect sizes were small, the LR test found more associations when effect sizes were larger, as it did also on real data. Second, we have developed computational speedups for both the LR and score tests—the former when the background kernel is full rank and the latter when the background and foreground kernels are low rank. For particularly onerous runs (e.g. gene–gene interaction set tests), where it may not be practical to run the more computationally expensive LR test, our efficient (and exact) score test can run about 2000 times faster on WTCCC data than score test algorithms currently available in statistical software, and ∼200 times faster than our efficient LR test.

One assumption underlying some of our efficient algorithms is that some of the kernels have a particular form (e.g. one which factors as an inner product, and generalizations of this). However, this assumption is reasonable, as it encompasses most of the kernels currently being used for set tests.

Directions for further consideration include incorporation and exploration of different measures of genetic similarity, and investigation of other score tests, beyond the one used here. Also, SKAT has been extended to encompass more ‘optimal’ settings of genetic similarity (Lee *et al.*, 2012a), for which our methods could likely be adapted. Another fruitful direction may be to use a hybrid LR-score approach for large datasets with a large number of hypotheses where the LR test may become impractical. For example, one might consider a full scan using the score test, and then scanning from the top of the resulting ranked list using the more powerful LR test, going as far down the list as resources permit. Modeling the null distribution of the score-based test statistic can be done in a variety of ways, including using Davies (1980) or Imhof’s method (Imhof, 1961) with a sum of $\chi_{1}$-distributed variables as we and SKAT have done, using Kuonen’s saddlepoint method (Chen *et al.*, 2013; Kuonen, 1999), or various types of moment matching (Li and Cui, 2012; Wu *et al.*, 2011). To our knowledge, a systematic comparison of these approaches has not been done, although, judging from our preliminary comparisons, we do not expect these variations to alter the overall story of how the LR test performs relative to the score test. Finally, further investigation into the robustness of each statistical test to model misspecification would be of practical interest.

## 4 METHODS

### 4.1 Statistical models and tests

Let N(u;Σ) denote a multivariate Normal distribution with mean u and covariance Σ. For the no-background-variance-component set test, the distribution of the phenotype is defined by a variance components model,

(1) $$y∼\text{N}\left(\beta X;\sigma_{e}^{2}I+\sigma_{1}^{2}K_{1}\right)$$

where y is a 1×N vector of phenotype values for N individuals; β (of dimension D×1) is the set of the D fixed effect for the covariates contained in the design matrix X; I is an N×N identity matrix; $\sigma_{e}^{2}$ is the residual variance; $K_{1}$, given by $K_{1}$ = $G_{1}G_{1}^{\text{T}}$, is a covariance matrix computed from the variants contained in the design matrix $G_{1}$ (dimension $N\times k_{1}$ and normalized for the number of SNPs in it) to be tested and has an associated variance $\sigma_{1}^{2}$.

Conditioned on the restricted maximum likelihood estimates for the nuisance parameter of fixed effects, β, the log restricted likelihood for model (1) is as follows
(2)
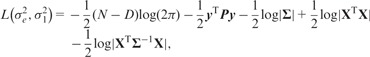

where the covariance $\Sigma \equiv (\sigma_{e}^{2}I+\sigma_{1}^{2}K_{1})$ is defined as in Equation (1) and where $P=(\Sigma^{-1}-\Sigma^{-1}X{\left(X^{T}\Sigma^{-1}X\right)}^{-1}X^{T}\Sigma^{-1})$ is a matrix that projects out the fixed effects.

When an additional background covariance matrix $K_{g}$ weighted by $\sigma_{g}^{2}$ is present (for example, to correct for confounding or include main effects in an interaction test), then the covariance is defined as $\Sigma \equiv (\sigma_{e}^{2}I+\sigma_{g}^{2}K_{g}+\sigma_{1}^{2}K_{1})$. The restricted log likelihood (2) forms the basis of both the score and LR tests, as described later and in the Supplementary Information.

The set test is formally defined as having a null hypothesis, $H_{0}:\sigma_{1}^{2}=0$, and alternative hypothesis $H_{1}:\sigma_{1}^{2}\geq 0$. In the statistical genetics community, this test is exclusively performed using a score test (Chen *et al.*, 2013; Ionita-Laza *et al.*, 2013; Lee *et al.*, 2012a; Listgarten *et al.*, 2013; Liu *et al.*, 2007, 2008; Oualkacha *et al.*, 2013; Schifano *et al.*, 2012; Wu *et al.*, 2011), except for (Listgarten *et al.*, 2013), which uses an LR test. For simplicity, we here describe the LR test for a single-variance component model, leaving the two-component model for the Supplementary Methods.

#### 4.1.1 One Variance Component Likelihood Ratio test

For the LR test, Equation (2) is maximized twice, once under the alternative hypothesis $H_{1}:\sigma_{1}^{2}\geq 0$, and once under the null hypothesis $H_{0}:\sigma_{1}^{2}=0$. Twice the difference in these maximum values is the LR test statistic. For this statistic, we assume a null distribution (Listgarten *et al.*, 2013) of the form $\pi \chi_{0}^{2}+\left(1-\pi \right)a\chi_{d}^{2}$, which is a mixture between a zero-degree of freedom χ^2^ distribution ($\chi_{0}^{2}$) and a scaled d-degree of freedom χ^2^ distribution, where d≥0 is a continuous number, scaled by a, and with mixture parameter π. To obtain the parameters of this null distribution, we permute the phenotype (and covariates) a small number of times (e.g. 10) to obtain a null distribution of test statistics. Using this empirical distribution, we then use a log-quantile regression of the top 10% of these to their theoretical expected values (conditioned on an estimate of π, which is estimated as the proportion of empirical null test statistics greater than zero). Now, given the fitted null distribution, we can apply it to the real distribution of test statistics to obtain *P*-values (Listgarten *et al.*, 2013). For the one-variance component model, the permutations can be done extremely cheaply by caching all the expensive computations from the real data (computations related to the matrix determinant and inverse), thereby reducing the computational complexity from quadratic to linear in the size of the gene sets.

#### 4.1.2 One variance component score test

For our one-variance component score test, we re-implemented the score-based approach, as used in SKAT (Wu *et al.*, 2011) (using equal weighting on all SNPs). The score-based test statistic, Q, is the phenotype-independent portion of the score (the first derivative of the restricted log likelihood (2) with respect to the parameter of interest, $\sigma_{1}^{2}$ as shown in the Supplementary Information) and is (in the one kernel case) given by the following:

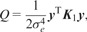

where $S\equiv I_{N}-X{\left(X^{T}X\right)}^{-1}X^{T}$. As in (Goeman *et al.*, 2006; Wu *et al.*, 2011) and as proven in the Supplementary Information, the distribution of Q under the null hypothesis is given by a weighted sum of one-degree of freedom $\chi^{2}$ variables,

$$Q∼\overset{N}{\underset{i=1}{\sum }}\phi_{i}\chi_{1,i}^{2},$$

where the weights, $\phi_{i}$, are given by the eigenvalues of $\frac{1}{2}P^{\frac{1}{2}}G_{1}G_{1}^{\text{T}}P^{\frac{1}{2}}$, where $P^{\frac{1}{2}}$ is any matrix square root of P. Given this distribution, several methods can be used to obtain its cumulative distribution, required for computing *P*-values. As in SKAT, we used the Davies exact method (Davies, 1980), which is exact up to numeric precision. The logistic version of the score-based test was re-implemented based on the Laplace approximation to the quasi likelihood, as used in (Breslow and Clayton, 1993). When $K=G_{1}G_{1}^{\text{T}}$ (such as when a linear covariance matrix in $G_{1}$ is used), one can implement both the one- and two-variance component tests efficiently.

Methods for two-component tests and for improving the computational efficiency of the LR and score tests are given in Supplementary Methods.

### 4.2 Datasets

The WTCCC 1 data (Burton *et al.*, 2007) consisted of the SNP and phenotype data for seven common diseases: bipolar disorder, coronary artery disease, hypertension, Crohn’s disease, rheumatoid arthritis, type-I diabetes (T1D) and type-II diabetes. Each phenotype group contained ∼1900 individuals. In addition, the data included a set of ∼1500 controls from the UK Blood Service Control Group (NBS). The data did not include a second control group from the 1958 British Birth Cohort (58C), as permissions for it precluded use by a commercial organization. SNPs were filtered more stringently than as described by the WTCCC so as to minimize the impact of assay artifacts. A SNP was excluded if its minor-allele frequency (MAF) was <1%, it was missing in >1% of individuals or its genetic distance was unknown. After filtering, 356 441 SNPs remained. Analysis of real data consisted of the controls and the cases for just the disease being analyzed, yielding roughly 3500 individuals for each analysis. Simulated WTCCC datasets were based on the SNP data for these individuals, and referred to as the common-variant setting.

All experiments for WTCCC used 13 850 gene sets (using any SNPs within the promoter or coding region of a gene). For the real data analysis, and synthetic power experiments, we used 10 permutations for the LR test approach (described next). For synthetic type I control experiments with the LR test, 72 runs of the 13 850 gene sets were performed, for 997 200 *P*-values generated from the null per method. A separate 10 permutations were used to fit the null distribution.

For the timing experiments on the WTCCC data, which used a two-variance component model, the dataset was augmented in several ways. First, the filtering of related individuals and different ancestral backgrounds was omitted, so that the two-variance component model would be needed. Second, for the analysis of a given phenotype, controls were taken to be the NBS controls as well as the cases for all other phenotypes. This setup resulted in 1984 T1D cases and 12 941 controls (and still 356 441 SNPs). Kernels with both all SNPs (full rank) and 150 SNPs (low rank case) were used.

For the experiments with rare variants, we used data from a BMI dataset (dbGap phs000169.v1.p1), which consisted of data for 2802 unrelated individuals from a 1 M Illumina chip. Keeping SNPs with MAF between 1 and 4% yielded 19 708 SNPs, from which 2030 non-singleton gene sets were formed (using any SNPs within the promoter or coding region of a gene). For the real data analysis and synthetic power experiments, we used 100 permutations for the LR test approach. For synthetic type I control experiments with the LR test, 500 runs of the 2030 gene sets were performed, for 1 015 000 *P*-values generated from the null per method. A separate 100 permutations were used to fit the null distribution.

The two datasets above were used both for analyzing their real data, and also using their real SNPs to generate synthetic phenotypes (as described below). In addition to these, when examining the performance of real datasets, we also analyzed a Warfarin phenotype dataset and an Atherosclerosis risk dataset.

The Warfarin dataset (Cooper *et al.*, 2008), processed as in (Tatonetti *et al.*, 2010), used LD thinning for SNPs with more than $r^{2}=0.2$, yielding 509 250 SNPs for 181 individuals, and 22 793 gene sets. The same gene sets as in (Tatonetti *et al.*, 2010) were used, which consisted of SNPs either contained within a given gene or within 5 kb upstream of downstream of that gene). Stable warfarin dosages were used as the phenotype. Covariates used in the analysis were sex, age, ancestral background, weight, treatment with amiodarone, and treatment with losartan. We used 10 permutations to get *P*-values for the LR test.

The ARIC Cohort (dbGaP Study Accession: phs000280.v2.p1) data were filtered as follows. First, any individual with more than 5% missing data was removed. Then, a SNP was excluded if its MAF was less than 1%, or it was missing in more than 2% of individuals. After processing, there were 12 751 individuals, 717 492 SNPs and 25 659 gene sets (using any SNPs within the coding region of a gene). We used age, sex and the community from which the individual came as covariates. We used eight of the ARIC phenotypes (trgsiu01, hdlsiu02, hd3siu02, hd2siu02, mi04, inc_by04, in_by04p, calc) that yielded similar genomic control factors (all less than 1.05) with and without a full background kernel to correct for population structure and family relatedness; these phenotypes also showed association signal (judging from the quantile–quantile plot of univariate –log(*P*) values. We used 10 permutations to get *P*-values for the LR test.

When a background kernel composed of all SNPs was used, we excluded any SNPs from the same chromosome as those being tested to avoid proximal contamination (Lippert *et al.*, 2011; Listgarten *et al.*, 2012). When the background kernel was low rank, we excluded any SNPs from the background kernel that were within 2 million bases of the SNPs being tested.

SNPs in all datasets, for both real and synthetic experiments, were encoded as 0,1,2 for the number of minor alleles, before being zero-meaned and whitened (SD set to 1.0).

### 4.3 Simulation experiments

All synthetic experiments comparing statistical tests used a standard variance components model in which there was only one kernel—the one used to test a set of SNPs, as in all of the SKAT papers (Lee *et al.*, 2012a and 2012b; Wu *et al.*, 2011). Real SNP data were used for the synthetic experiments, and only the phenotype was simulated. For experiments measuring control of type I error, continuous phenotypes were sampled independently from a Gaussian distribution and, therefore, contained no genetic signal. Binary phenotypes were obtained by thresholding the Gaussian phenotype such that the case:control ratio was 50:50. For power experiments, a new phenotype was sampled for each gene in turn, using all SNPs in that gene, over a variety of effect strengths. The phenotype was sampled from the LMM, using $\sigma_{g}^{2}=1.0,0.1,0.01,0.001$ and $\sigma_{e}^{2}=1.0$, which is equivalent to sampling from a linear regression fixed effects model (Listgarten *et al.*, 2012) with noise $\sigma_{e}^{2}$ and fixed effect weight parameters identically and independently distributed from a Gaussian with variance $\sigma_{g}^{2}$. In the text, we often describe these relative effect sizes using 
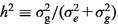
. For each value of $\sigma_{g}^{2}$, five random seeds were used to generate five phenotypes.

To examine robustness to model misspecification (which was not present in the setting just described), we also altered the experimental set-up above, by variously using each of these, independently:

1. using all SNPs on all even-numbered chromosomes (in WTCCC) to generate a background polygenic signal with SNP effects drawn from a zero-mean Gaussian with variance 1.0 (and no other noise), and then testing each gene set on only the odd-numbered chromosomes, having generated either with no additional phenotypic signal (for type 1 error), or having added in gene-set specific effects (for all SNPs) drawn from a zero-mean Gaussian with variance set to each of $10^{-4},10^{-3},10^{-2},10^{-1}$, or
2. the same as above, but using only 50, 10 or 1% of the SNPs in a gene as causal for the power experiments.

## Supplementary Material

Supplementary Data
