# Supplementary material for: Greater power and computational efficiency for kernel-based association testing of sets of genetic variants
Source: Bioinformatics. 2014 Jul 29;30(22):3206–14. doi: 10.1093/bioinformatics/btu504 (PMC4221116; doi:10.1093/bioinformatics/btu504)
Supplement: Supplementary Data [file supp_30_22_3206__index.html]

Greater power and computational efficiency for kernel-based association testing of sets of genetic variants — Greater power and computational efficiency for kernel-based association testing of sets of genetic variants — Supplementary Data 

# Greater power and computational efficiency for kernel-based association testing of sets of genetic variants

## Supplementary Data

file

**Files in this Data Supplement:**

- Supplementary Data - pdf file
