# Supplementary material for: Greater power and computational efficiency for kernel-based association testing of sets of genetic variants
Source: Bioinformatics. 2014 Jul 29;30(22):3206–14. doi: 10.1093/bioinformatics/btu504 (PMC4221116; doi:10.1093/bioinformatics/btu504)
Supplement: Supplementary Data [file supp_btu504_mergedSI.pdf]

SUPPLEMENTARY FIGURES , TABLES AND METHODS FOR:

Greater Power and Computational Efficiency for Kernel-Based Association Testing of Sets of Genetic Variants

Christoph Lippert , Jing Xiang, Danilo Horta, Christian Widmer, Carl Kadie, David Heckerman, Jennifer Listgarten

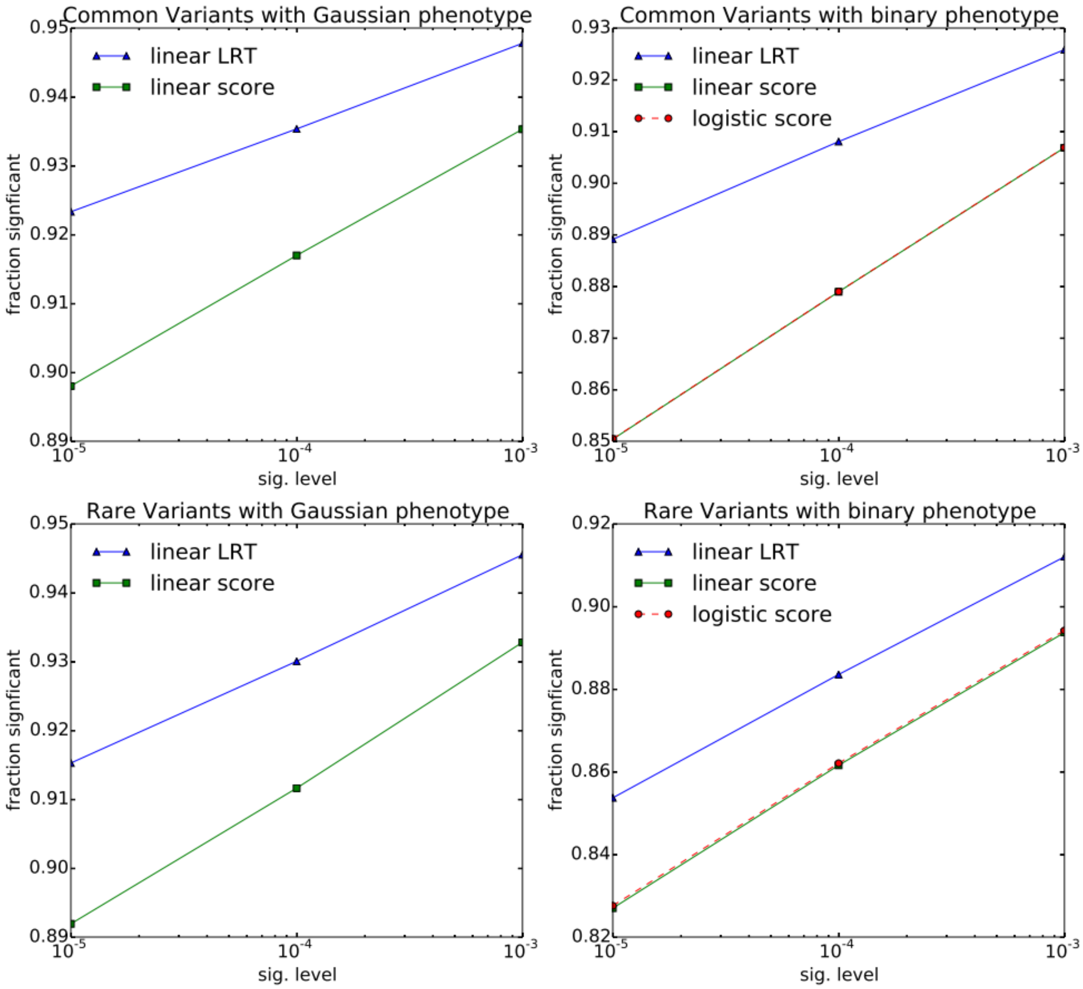

Supplementary Figure 1. Power on synthetic data for each method in each setting, for the lowest signal strength, $h^2 = 0.1$. Fraction of tests deemed significant across various significance levels for each method is shown on the y axis. Threshold for significance shown on the *x* axis. Other signal strengths are shown in Figures 1 and 2 and in Supplementary Figure 2.

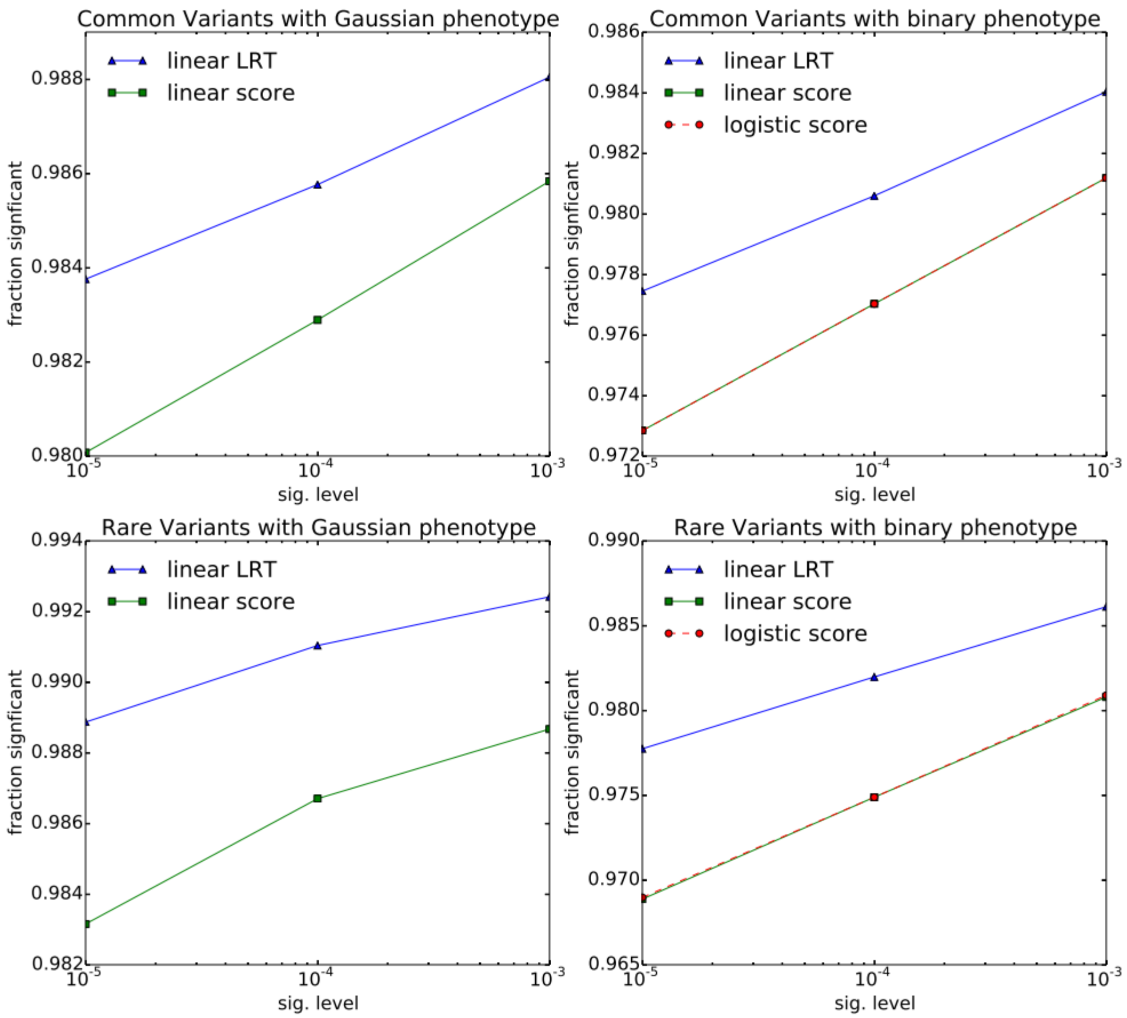

Supplementary Figure 2. Power on synthetic data for each method in each setting, for the lowest signal strength, $h^2 = 0.5$. Fraction of tests deemed significant across various significance levels for each method is shown on the y axis. Threshold for significance shown on the *x* axis. Other signal strengths are shown in Figures 1 and 2 and in Supplementary Figure 2.

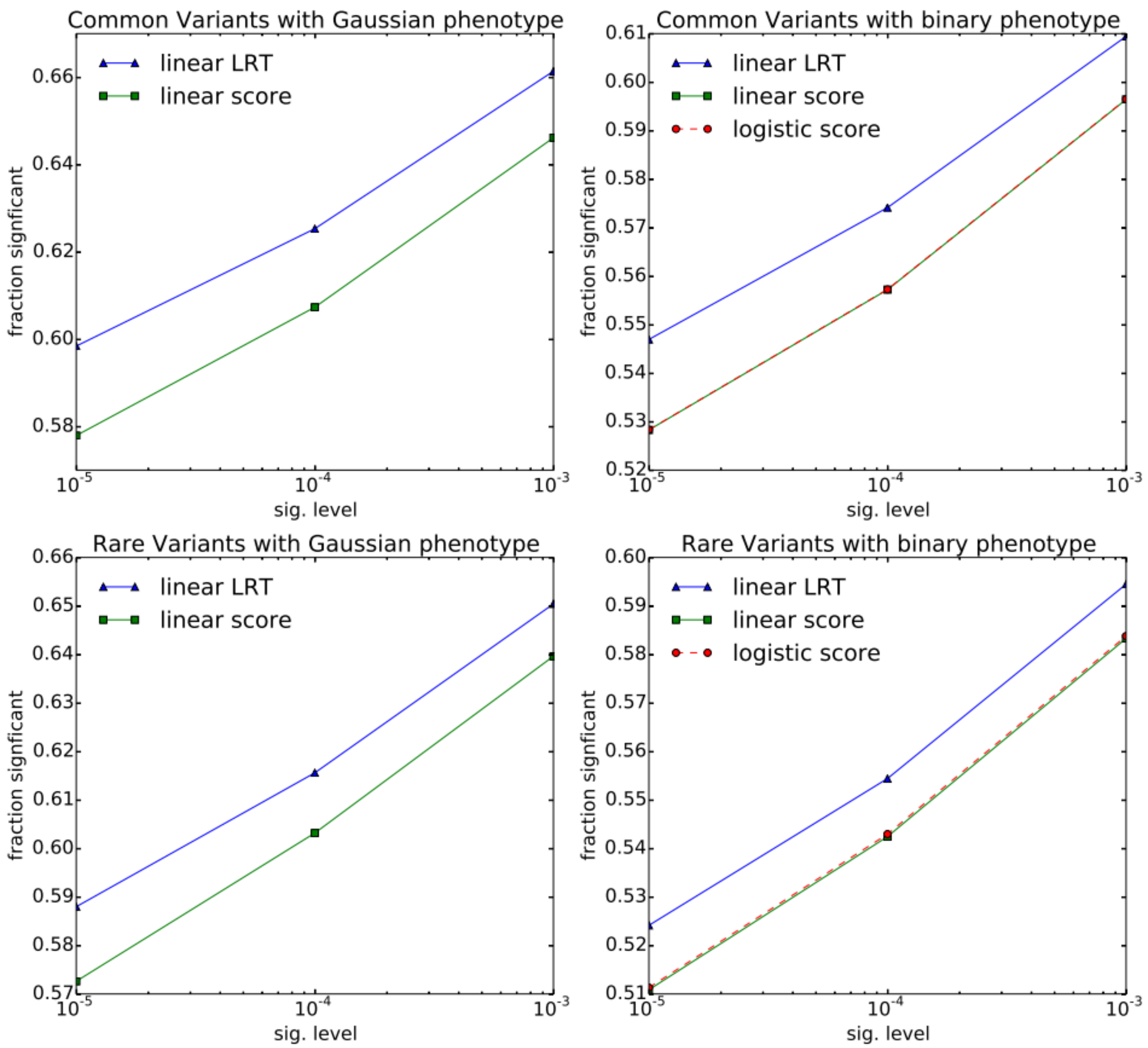

Supplementary Figure 3. Power on synthetic data for each method in each setting, aggregated across all signal strengths, $h^2 = 0.001, 0.01, 0.1, 0.5$. Fraction of tests deemed significant across various significance levels for each method is shown on the y axis. Threshold for significance shown on the *x* axis. Other signal strengths are shown in Figures 1 and 2 and in Supplementary Figures 1 and 2.

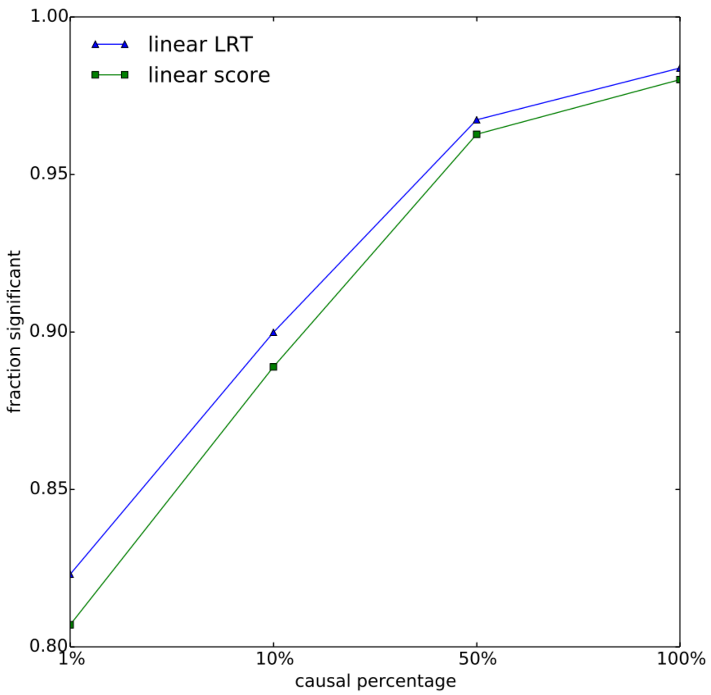

Supplementary Figure 4. Relative power gain on synthetic data for the LR over the score test decreases as more and more SNPs in a gene set become causal (from 1% to 100%), reflective of less and less model misspecification (with none at 100%). Shown is for α=1e-5, although a similar trend is observed for other significance thresholds. Strength of the gene set signal here was $h^2 = 50\%$.

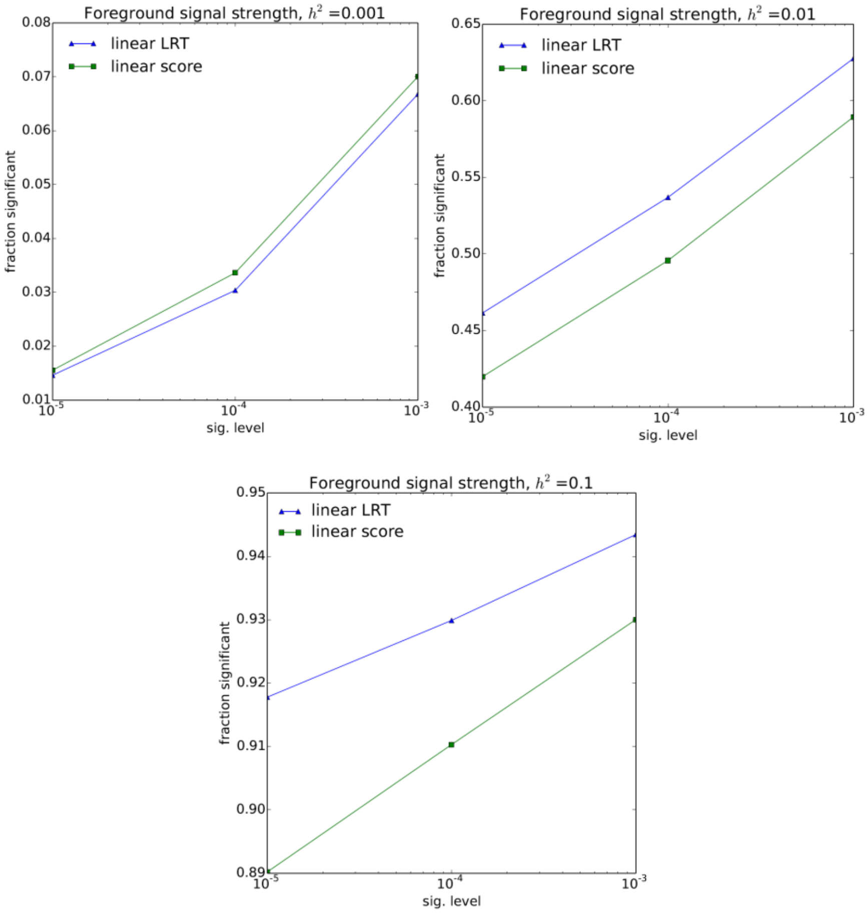

Supplementary Figure 5. Power experiments on synthetic data under model misspecification wherein a large polygenic background signal is used for the noise. The background signal was from all even chromosome SNPs, and then we tested only sets from odd chromosomes (and injected corresponding signal for each respective SNP set). The background noise had a variance of 1.0, while the foreground (set) variance was 0.001, 0.01, and 0.1, yielding $h^2 = 0.001, 0.01, 0.1$. The relative gain in fraction of significant hits decreased with increasing foreground signal strength. Equivalently, the relative gain in fraction of significant hits increased with increasing polygenic signal strength. For example, at a foreground signal strength of 0.01, the relative percent gain was 12% ((46-41)/41*100), while at a foreground signal strength of 0.1, the relative percent gain was only 3% ((92-89)/89*100).

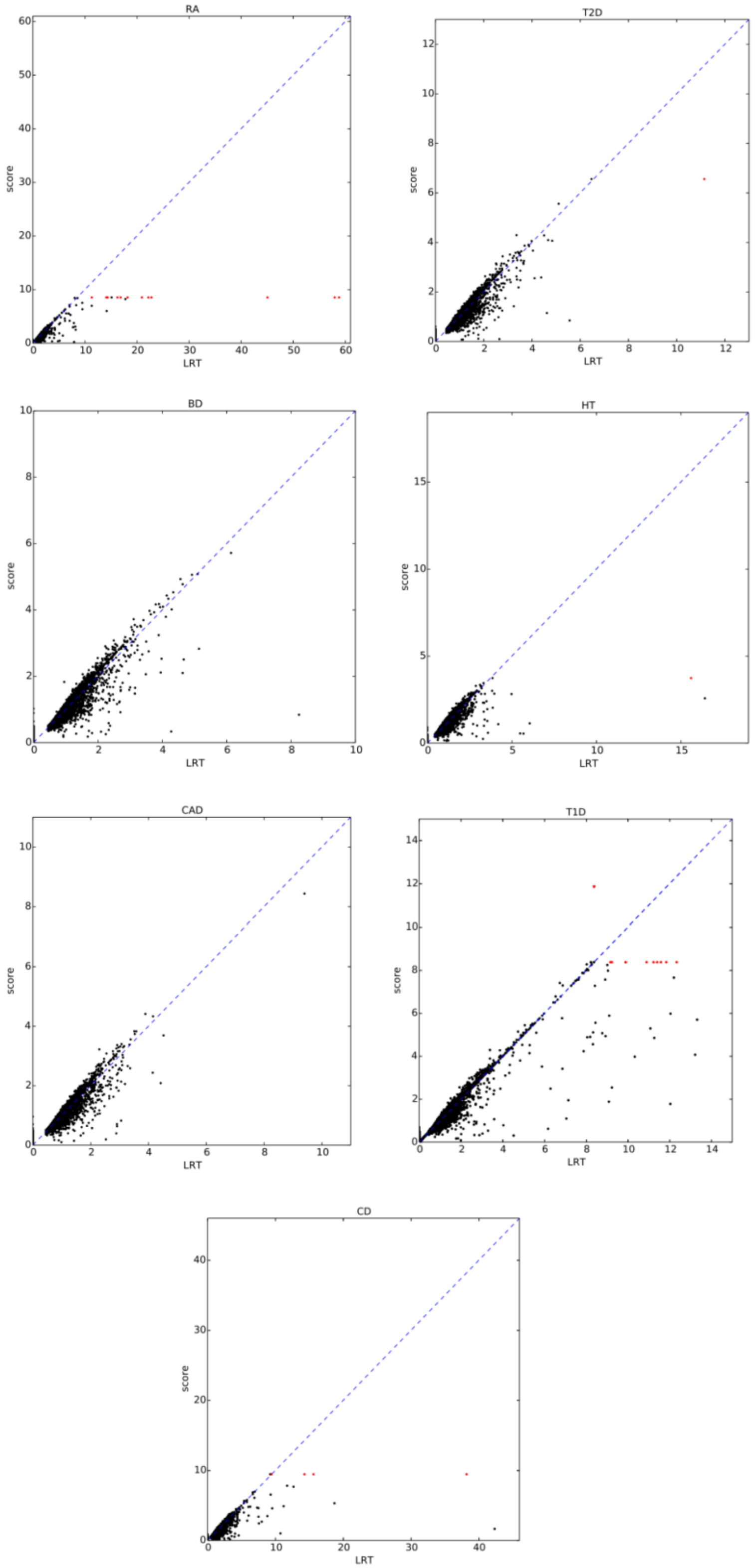

Supplementary Figure 6. Paired plots of the $-\log_{10} p$ values from the analysis of the seven WTCCC phenotypes in Table 3 of the main paper (with no background variance component). Red points denote hypotheses for which the numerical routine to compute the p-value for the score test lost precision and returned p=0. For these points, we assigned them a score p-value equal to the lowest observed p-value in that experiment.

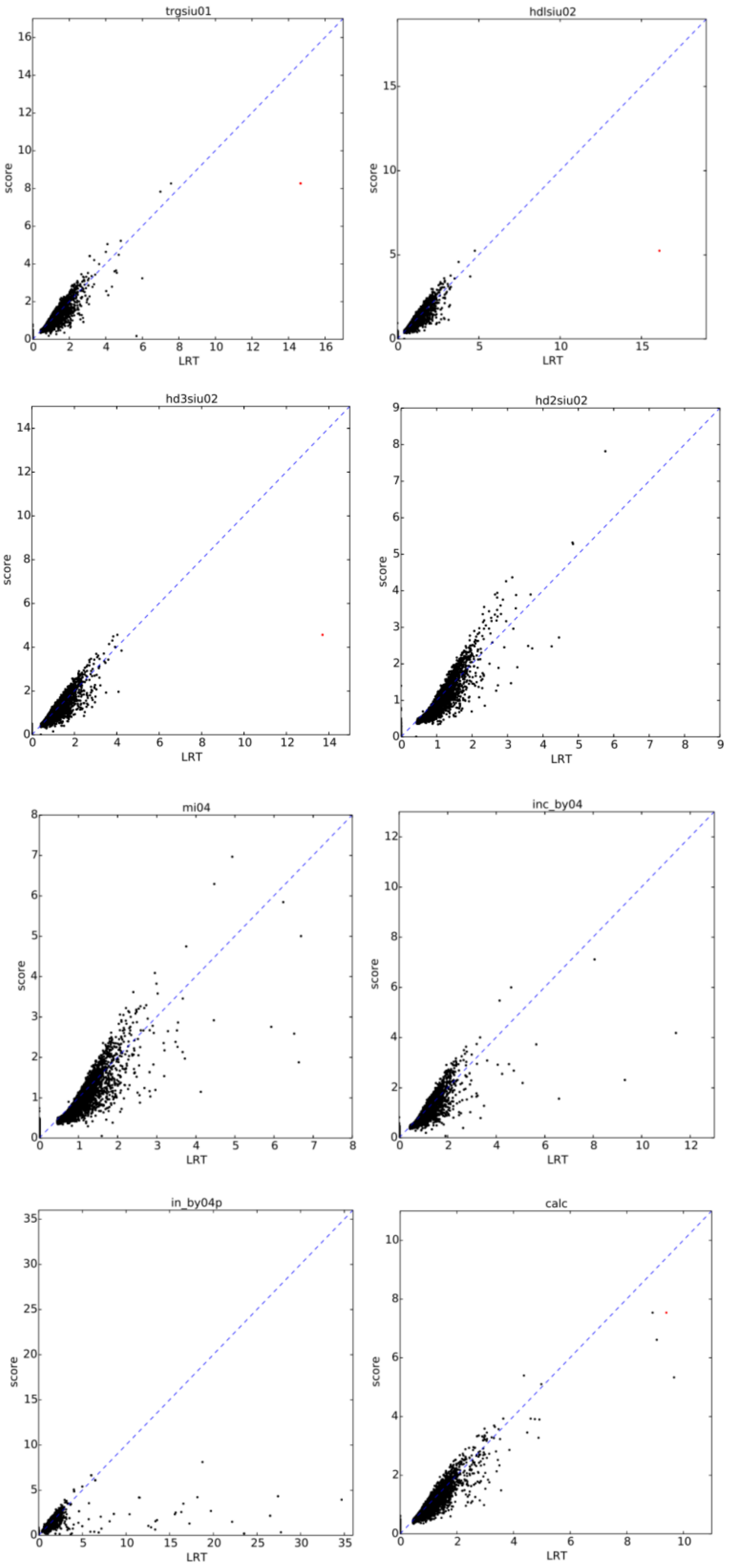

Supplementary Figure 7. Paired plots of the $-\log_{10} p$ values from the analysis of the eight ARIC phenotypes in Table 3 of the main paper (using no background variance component). Red points denote hypotheses for which the numerical routine to compute the p-value for the score test lost precision and returned p=0. For these points, we assigned them a score p-value equal to the lowest observed p-value in that experiment.

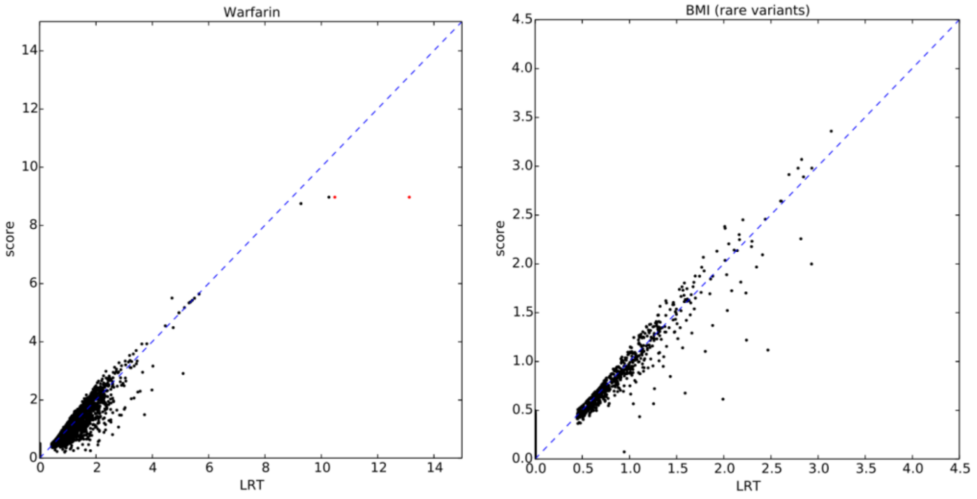

Supplementary Figure 8. Paired plots of the $-\log_{10} p$ values from the analysis of the Warfarin and BMI (with rare variants) phenotypes in Table 3 of the main paper (no background variance component used). Red points denote hypotheses for which the numerical routine to compute the p-value for the score test lost precision and returned p=0. For these points, we assigned them a score p-value equal to the lowest observed p-value in that experiment.

Supplementary Table 1. Type I error for WTCCC data using half of chromosomes as polygenic background.

**Gaussian phenotype**	$\alpha = 10^{-5}$	$\alpha = 10^{-4}$	$\alpha = 10^{-3}$
Score	1.1×10^{-5}	1.2×10^{-4}	1.0×10^{-3}
Linear LR test	7.9×10^{-6}	1.2×10^{-4}	1.0×10^{-3}

There are no statistically significant deviations from expectation according to binomial test with significance level of 0.05.

Supplementary Table 2. Validation of the three immune-related phenotypes from the WTCCC analysis. Raw number of validated hits found by each method. Data for validation were downloaded on 5/27/2014 from http://immunobase.org. "Max # could validate" denotes the total number of genes in our analysis found in the immune database, while, "# in db" denotes the total number genes listsed in the immune database. "1K" denotes the analysis with no background variance component, while "2K" denotes the analysis with a background variance component, as described in the main paper.

**data set**	**phenotype**	**Score (1K)**	**LRT (1K)**	**Score (2K)**	**LRT (2K)**	**Max # could validate**	**# in db**
WTCCC	CD	14	25	3	3	492	1045
WTCCC	T1D	36	44	32	42	207	481
WTCCC	RA	24	26	21	25	177	401

Supplementary Methods

1 Definitions and frequently used identities

- $\boldsymbol{I}_J$ denotes a J-by-J identity matrix.
- $\boldsymbol{I}$ denotes an identity matrix, where the dimensionality follows from the context.
- N-by-1 vector of phenotypes $\boldsymbol{y}$
- N-by-D covariates matrix $\boldsymbol{X}$ with full column rank D and $N \geq D$
- N-by-N covariance matrix $\boldsymbol{\Sigma_\theta}$, for the Gaussian-distributed phenotype $\boldsymbol{y}$, parameterized by the vector of covariance parameters $\boldsymbol{\theta} \in \boldsymbol{\Theta}$. In this paper we assume a simple weighted sum of fixed individual positive semi-definite matrices. So in the case of no background kernel (that is, no kernel appearing in the null model), $\boldsymbol{\Sigma_\theta} = \sigma_{\mathrm{e}}^2\boldsymbol{I} + \sigma_1^2\boldsymbol{K}_1$, whereas for the case where there is a background kernel, $\boldsymbol{\Sigma_\theta} = \sigma_{\mathrm{e}}^2\boldsymbol{I} + \sigma_{\mathrm{g}}^2\boldsymbol{K}_g + \sigma_1^2\boldsymbol{K}_1$. In both cases, under the null hypothesis $\sigma_1^2 = 0$, as this is the parameter being tested. $\boldsymbol{\Sigma_\theta}$ is assumed to have full rank N (which it will so long as $\sigma_{\mathrm{e}}^2 > 0$).
- N-by-N symmetric covariate orthogonal projection matrix $\boldsymbol{S} = \left(\boldsymbol{I}_N - \boldsymbol{X}\left(\boldsymbol{X}^\top\boldsymbol{X}\right)^{-1}\boldsymbol{X}^\top\right)$ and has rank $N - D$.
- N-by-N symmetric matrix $\boldsymbol{P_\theta} = \left(\boldsymbol{\Sigma_\theta}^{-1} - \boldsymbol{\Sigma_\theta}^{-1}\boldsymbol{X}(\boldsymbol{X}^{\mathrm{T}}\boldsymbol{\Sigma_\theta}^{-1}\boldsymbol{X})^{-1}\boldsymbol{X}^\top\boldsymbol{\Sigma_\theta}^{-1}\right)$ and has rank $N - D$.
- For any I-by-J matrix $\boldsymbol{A}$, the J-by-I matrix $\boldsymbol{A}^\dagger$ denotes the Moore-Penrose pseudo-inverse of $\boldsymbol{A}$.
- For any square I-by-I matrix $\boldsymbol{A}$, the expression $|\boldsymbol{A}|_+$ denotes the pseudo-determinant of $\boldsymbol{A}$. If $\boldsymbol{A}$ is positive semi-definite, then $|\boldsymbol{A}|_+$ may be computed as the product of the non-zero eigenvalues. If all eigenvalues are zero, then the pseudo-determinant is 1.
- $\boldsymbol{SS} = \boldsymbol{S}$. (See Proposition 5)
- $\boldsymbol{SX} = \boldsymbol{0}$. (See Proposition 4)
- $\boldsymbol{P_\theta X} = \boldsymbol{0}$. (See Proposition 6)
- $\boldsymbol{P_\theta S} = \boldsymbol{P_\theta} = \boldsymbol{SP_\theta}$. (See Proposition 7)
- $\boldsymbol{P_\theta}^\dagger = \boldsymbol{S\Sigma_\theta S}$ and equivalently $\boldsymbol{P_\theta} = \left(\boldsymbol{S\Sigma_\theta S}\right)^\dagger$ (See Lemma 12)
- For any I-by-J matrix $\boldsymbol{A}$ and any parameter θ, $\frac{\partial \boldsymbol{A}}{\partial \theta}$ is the I-by-J matrix derivative of $\boldsymbol{A}$ with respect to any covariance parameter θ.
- $\frac{\partial \boldsymbol{P_\theta}}{\partial \theta_i} = -\boldsymbol{P_\theta}\frac{\partial \boldsymbol{P_\theta}}{\partial \theta_i}\boldsymbol{P_\theta}$ (See Proposition 8)
- $\mathrm{Tr}(\boldsymbol{AB}) = \mathrm{Tr}(\boldsymbol{BA})$ for any two matrices $\boldsymbol{A}$ and $\boldsymbol{B}$.
- $\boldsymbol{A}_\perp$ is a matrix with columns that are all orthogonal to the columns of the matrix $\boldsymbol{A}$.

2 Restricted Maximum Likelihood (REML)

For REML estimation [7] of the mixed model variance parameters, we use the restricted log likelihood [3] for N individuals with the N-by-1 vector of phenotypes $\boldsymbol{y}$, the N-by-D matrix of covariates $\boldsymbol{X}$ and the covariance matrix $\boldsymbol{\Sigma_\theta}$, parameterized by $\boldsymbol{\theta} \in \boldsymbol{\Theta}$, given by

$$\mathcal{L}(\boldsymbol{\theta}) = -\frac{(N-D)}{2}\log(2\pi) - \frac{1}{2}\boldsymbol{y}^{\mathrm{T}}\boldsymbol{P_\theta y} - \frac{1}{2}\log|\boldsymbol{\Sigma_\theta}| + \frac{1}{2}\log|\boldsymbol{X}^{\mathrm{T}}\boldsymbol{X}| - \frac{1}{2}\log|\boldsymbol{X}^{\mathrm{T}}\boldsymbol{\Sigma_\theta}^{-1}\boldsymbol{X}|. \quad (1)$$

Using Proposition 19, the restricted likelihood can be simplified using $\boldsymbol{P_\theta}$ only.

$$\mathcal{L}(\boldsymbol{\theta}) = -\frac{(N-D)}{2}\log(2\pi) - \frac{1}{2}\boldsymbol{y}^{\mathrm{T}}\boldsymbol{P_\theta y} + \frac{1}{2}\log|\boldsymbol{P_\theta}|_{+}. \quad (2)$$

3 Variance component tests

We perform variance component tests, by modifying the matrix $\boldsymbol{\Sigma_\theta}$ (and consequently the matrix $\boldsymbol{P_\theta}$) with respect to the variance parameters σ_{e}^2, σ_{g}^2 and σ_1^2, and the kernel matrices $\boldsymbol{K}_1$ and $\boldsymbol{K}_g$.

Recall that in the case of a variance component test without a background kernel (that is, no kernel $\boldsymbol{K}_g$ in the null model) $\boldsymbol{\Sigma_\theta} = \sigma_{\mathrm{e}}^2\boldsymbol{I} + \sigma_1^2\boldsymbol{K}_1$, whereas for the case where there is a background kernel, $\boldsymbol{\Sigma_\theta} = \sigma_{\mathrm{e}}^2\boldsymbol{I} + \sigma_{\mathrm{g}}^2\boldsymbol{K}_g + \sigma_1^2\boldsymbol{K}_1$. In both cases, under the null hypothesis we set the parameter of interest σ_1^2 to 0 and perform REML estimation on the remaining parameters only.

4 Efficient likelihood ratio tests in the presence of full-rank background kernels

In [6] it was shown how the likelihood of the mixed model can efficiently be maximized, when both $\boldsymbol{K}_g$ and $\boldsymbol{K}_1$ are low rank and can be factored, respectively, as $\boldsymbol{G}_g\boldsymbol{G}_g^\top$ and $\boldsymbol{G}_1\boldsymbol{G}_1^\top$. Here, we make no special assumptions (e.g. rank) on the background kernel $\boldsymbol{K}_g$, only assuming that the variance component $\boldsymbol{K}_1$ (the component being tested, corresponding to, say, variants in a gene set) is low rank and can be factored as $\boldsymbol{G}_1\boldsymbol{G}_1^\top$.

$$\boldsymbol{\Sigma_\theta} = \sigma_{\mathrm{e}}^2\boldsymbol{I} + \sigma_{\mathrm{g}}^2\boldsymbol{K}_g + \sigma_1\boldsymbol{G}_1\boldsymbol{G}_1^T.$$

Introducing $\delta = \sigma_{\mathrm{e}}^2/\sigma_{\mathrm{g}}^2$ and $\gamma_1 = \sigma_1^2/\sigma_{\mathrm{g}}^2$, we obtain

$$\boldsymbol{\Sigma_\theta} = \sigma_{\mathrm{g}}^2\left(\delta\boldsymbol{I} + \boldsymbol{K}_g - \gamma_1^2\boldsymbol{G}_1\boldsymbol{G}_1^T\right).$$

There are several variations of this basic setting that we want to handle efficiently (and do handle efficiently). These cases are as follows.

The first case occurs when there are variants, $\boldsymbol{W}_g$, among the variants we are testing ($\boldsymbol{G}_1$), which are also used in construction of the background kernel, $\boldsymbol{K}_g$. In this setting, we wish to remove these variants from the background kernel, in order to correct for proximal contamination [4, 5]) without explicitly computing a new background kernel from scratch.

As $\boldsymbol{K}_g$ decomposes in a sum over contributions from individual variants, we can simply subtract off the contribution of $\boldsymbol{W}_g$ from the complete matrix $\boldsymbol{K}_g$ [5].

$$\boldsymbol{\Sigma_\theta} = \sigma_{\mathrm{g}}^2 \left(\delta \boldsymbol{I} + \boldsymbol{K}_g - \boldsymbol{W}_g \boldsymbol{W}_g^\top + \gamma_1^2 \boldsymbol{G}_1 \boldsymbol{G}_1^T\right).$$

A special case of this occurs when the set of variants being tested $\boldsymbol{G}_1$ is a subset of the set being removed from the genetic background kernel. In this case, one can remove this subset from the matrix $\boldsymbol{W}_g$ and instead modify the variance parameters as follows for additional computational savings:

$$\boldsymbol{\Sigma_\theta} = \sigma_{\mathrm{g}}^2 \left(\delta \boldsymbol{I} + \boldsymbol{K}_g - \boldsymbol{W}_g \boldsymbol{W}_g^\top + (\gamma_1^2 - 1) \boldsymbol{G}_1 \boldsymbol{G}_1^T\right).$$

All of these cases can be treated by aggregating all of the ”updating factors” (those factors which are added/subtracted from $\boldsymbol{K}_g$) into a single N-by-k the matrix $\boldsymbol{W}$ and maintaining their relative ”weighting” (i.e., variance parameters) by additionally introducing the k-by-k diagonal weight matrix $\boldsymbol{\Gamma}$, which contains the variance parameters for each column in $\boldsymbol{W}$ on the diagonal,

$$\boldsymbol{\Sigma_\theta} = \sigma_{\mathrm{g}}^2 \left(\delta \boldsymbol{I} + \boldsymbol{K}_g + \boldsymbol{W} \boldsymbol{\Gamma} \boldsymbol{W}^\top\right).$$

Now that we have presented the cases of interest, we next show how to perform efficient computations for these cases by using the idea of low-rank updates [5] to the full rank matrix $\boldsymbol{K}_g$. In Sections 4.1.1 and 4.1.2 we provide the updated squared form part and the updated determinant part of the REML likelihood respectively.

4.1 Low rank update

The computational bottleneck of the likelihood ratio test is computation of the restricted log likelihood (Equation 2), which contains several expensive terms if computed naively (the squared form, $\frac{1}{2}\boldsymbol{y}^\mathrm{T}\boldsymbol{P_\theta}\boldsymbol{y}$, and the pseudo determinant of $\boldsymbol{P_\theta}$, $\frac{1}{2}\log|\boldsymbol{P_\theta}|_+$. Next we show how to compute each of them efficiently (in terms of time).

$$\begin{aligned}
\boldsymbol{P_\theta} &= (\boldsymbol{S}\boldsymbol{\Sigma_\theta}\boldsymbol{S})^\dagger \\
&= \left(\boldsymbol{S}\sigma_{\mathrm{g}}^2 \left(\delta \boldsymbol{I} + \boldsymbol{K}_g + \boldsymbol{W}\boldsymbol{\Gamma}\boldsymbol{W}^\top\right)\boldsymbol{S}\right)^\dagger \\
&= \sigma_{\mathrm{g}}^{-2}\left(\boldsymbol{S}\left(\delta\boldsymbol{I} + \boldsymbol{K}_g\right)\boldsymbol{S} + \boldsymbol{S}\boldsymbol{W}\boldsymbol{\Gamma}\boldsymbol{W}^\top\boldsymbol{S}\right)^\dagger \\
&= \sigma_{\mathrm{g}}^{-2}\left(\boldsymbol{U}\left(\delta\boldsymbol{I} + \boldsymbol{\Lambda}\right)\boldsymbol{U}^\top + \boldsymbol{U}\boldsymbol{U}^\top\boldsymbol{W}\boldsymbol{\Gamma}\boldsymbol{W}^\top\boldsymbol{U}\boldsymbol{U}^\top\right)^\dagger \\
&= \sigma_{\mathrm{g}}^{-2}\left(\boldsymbol{U}\left(\delta\boldsymbol{I} + \boldsymbol{\Lambda} + \boldsymbol{U}^\top\boldsymbol{W}\boldsymbol{\Gamma}\boldsymbol{W}^\top\boldsymbol{U}\right)\boldsymbol{U}^\top\right)^\dagger \\
&= \sigma_{\mathrm{g}}^{-2}\boldsymbol{U}\left(\delta\boldsymbol{I} + \boldsymbol{\Lambda} + \boldsymbol{U}^\top\boldsymbol{W}\boldsymbol{\Gamma}\boldsymbol{W}^\top\boldsymbol{U}\right)^{-1}\boldsymbol{U}^\top \\
&= \sigma_{\mathrm{g}}^{-2}\boldsymbol{U}\left(\delta\boldsymbol{I} + \boldsymbol{\Lambda}\right)^{-1}\boldsymbol{U}^\top \\
&\quad - \sigma_{\mathrm{g}}^{-2}\boldsymbol{U}\left(\delta\boldsymbol{I} + \boldsymbol{\Lambda}\right)^{-1}\boldsymbol{U}^\top\boldsymbol{W}\left(\boldsymbol{\Gamma}^{-1} + \boldsymbol{W}^\top\boldsymbol{U}\left(\delta\boldsymbol{I} + \boldsymbol{\Lambda}\right)^{-1}\boldsymbol{U}^\top\boldsymbol{W}\right)^{-1}\boldsymbol{W}^\top\boldsymbol{U}\left(\delta\boldsymbol{I} + \boldsymbol{\Lambda}\right)^{-1}\boldsymbol{U}^\top
\end{aligned} \tag{3}$$

Assuming that the eigenvalue decomposition of $\boldsymbol{S}\left(\delta\boldsymbol{I} + \boldsymbol{K}_g\right)\boldsymbol{S}$ has been pre-computed, the additional computations required are multiplication of $\boldsymbol{W}$ with the matrix $\boldsymbol{U}$, an $O(N^2k)$ operation. Once this has been computed once for the corresponding matrix $\boldsymbol{W}$, the remaining computations can be performed in $O(Nk^2)$.

4.1.1 Squared form update

Given the derivation of the update on $\boldsymbol{P_\theta}$, we can efficiently plug this into the squared form.

$$\begin{aligned}\frac{1}{2}\boldsymbol{y}^{\mathrm{T}}\boldsymbol{P_\theta}\boldsymbol{y} =& \frac{1}{2\sigma_{\mathrm{g}}^2}\boldsymbol{y}^{\mathrm{T}}\boldsymbol{U}\left(\delta\boldsymbol{I}+\boldsymbol{\Lambda}\right)^{-1}\boldsymbol{U}^{\top}\boldsymbol{y} \\ &- \frac{1}{2\sigma_{\mathrm{g}}^2}\boldsymbol{y}^{\mathrm{T}}\boldsymbol{U}\left(\delta\boldsymbol{I}+\boldsymbol{\Lambda}\right)^{-1}\boldsymbol{U}^{\top}\boldsymbol{W}\left(\boldsymbol{\Gamma}^{-1}+\boldsymbol{W}^{\top}\boldsymbol{U}\left(\delta\boldsymbol{I}+\boldsymbol{\Lambda}\right)^{-1}\boldsymbol{U}^{\top}\boldsymbol{W}\right)^{-1}\boldsymbol{W}^{\top}\boldsymbol{U}\left(\delta\boldsymbol{I}+\boldsymbol{\Lambda}\right)^{-1}\boldsymbol{U}^{\top}\boldsymbol{y}\end{aligned}$$

4.1.2 Determinant update

For the determinant we need to modify the low rank update a bit in order to avoid numerical instability. The problem is that the update matrix is not necessarily positive-semi-definite.

$$\begin{aligned}\frac{1}{2}\log|\boldsymbol{P_\theta}|_+ &= \frac{1}{2}\log|\boldsymbol{U}^{\top}\boldsymbol{\Sigma_\theta}\boldsymbol{U}|_+ && (4)\\ &= \frac{1}{2}\log|\sigma_{\mathrm{g}}^2\left(\delta\boldsymbol{I}_{N-D}+\boldsymbol{\Lambda}+\underbrace{\boldsymbol{U}^{\top}\boldsymbol{W}\boldsymbol{\Gamma}}_{\boldsymbol{A}}\underbrace{\boldsymbol{W}^{\top}\boldsymbol{U}}_{\boldsymbol{B}^{\top}}\right)| && (5)\\ &= \frac{N-D}{2}\log\sigma_{\mathrm{g}}^2+\frac{1}{2}\log|\delta\boldsymbol{I}_{N-D}+\boldsymbol{\Lambda}+\boldsymbol{A}\boldsymbol{B}^{\top}| && (6)\\ &= \frac{N-D}{2}\log\sigma_{\mathrm{g}}^2+\frac{1}{2}\log\left(|\delta\boldsymbol{I}_{N-D}+\boldsymbol{\Lambda}|\cdot|\boldsymbol{I}+\boldsymbol{B}^{\top}\left(\delta\boldsymbol{I}_{N-D}+\boldsymbol{\Lambda}\right)^{-1}\boldsymbol{A}|\right), && (7)\\ &= \frac{N-D}{2}\log\sigma_{\mathrm{g}}^2+\frac{1}{2}\log\left(|\delta\boldsymbol{I}_{N-D}+\boldsymbol{\Lambda}|\right)+\log\left(|\boldsymbol{I}+\boldsymbol{B}^{\top}\left(\delta\boldsymbol{I}_{N-D}+\boldsymbol{\Lambda}\right)^{-1}\boldsymbol{A}|\right), && (8)\end{aligned}$$

where we have used Sylvester's theorem for matrix determinants, which states that for matrices $\boldsymbol{A}$ and $\boldsymbol{B}$ respectively of size, $p\times n$ and $n\times p$, that

$$|\boldsymbol{I}_p+\boldsymbol{A}\boldsymbol{B}|=|\boldsymbol{I}_n+\boldsymbol{B}\boldsymbol{A}|. \tag{9}$$

For numerical reasons it is preferable to avoid computation of any matrix determinants and instead diagonalize the matrix its eigenvalue decomposition and adding up the logarithms of the respective eigenvalues. Yet the matrix $\boldsymbol{A}\boldsymbol{B}^{\top}$ will have negative eigenvalues if there is at least one negative diagonal entry in $\boldsymbol{\Gamma}$, such that that we cannot just take logarithms of its eigenvalues.

Proposition 1. *The matrix* $\boldsymbol{I}+\boldsymbol{B}^{\top}\left(\delta\boldsymbol{I}_{N-D}+\boldsymbol{\Lambda}\right)^{-1}\boldsymbol{A}$ *has an even number of negative eigenvalues.*

Proof. By construction we know that the matrix $\boldsymbol{P_\theta}$ is positive semi-definite, implying that its pseudo-determinant is positive. As the matrix $\delta\boldsymbol{I}_{N-D}+\boldsymbol{\Lambda}$ also is positive definite, implying that its determinant is positive, it follows that $\boldsymbol{A}\boldsymbol{B}^{\top}$ must also have a positive determinant. As the determinant equals the product of the eigenvalues, positivity of the determinant implies that the number of negative eigenvalues must be even. □

As a result we can compute the log determinant as the sum of the logarithms of the absolute values of the eigenvalues and avoid taking logarithms of any negative numbers.

The computational complexity for computing the determinant update equals that for the squared form update.

5 Score-based test

We here define the score function of any given covariance parameter, θ_i, as the derivative of the log restricted likelihood in Equation 1 with respect to θ_i

$$
\begin{aligned}
\frac{\partial \mathcal{L}(\boldsymbol{\theta})}{\partial \theta_i} &= -\frac{1}{2}\frac{\partial \boldsymbol{y}^{\mathrm{T}}\boldsymbol{P_\theta}\boldsymbol{y}}{\partial \theta_i} - \frac{1}{2}\frac{\partial \log|\boldsymbol{\Sigma_\theta}|}{\partial \theta_i} - \frac{1}{2}\frac{\partial \log|\boldsymbol{X}^{\mathrm{T}}\boldsymbol{\Sigma_\theta}^{-1}\boldsymbol{X}|}{\partial \theta_i} \\
&= -\frac{1}{2}\boldsymbol{y}^\top\frac{\partial \boldsymbol{P_\theta}}{\partial \theta_i}\boldsymbol{y} - \frac{1}{2}\operatorname{Tr}\left(\boldsymbol{\Sigma_\theta}^{-1}\frac{\partial \boldsymbol{\Sigma_\theta}}{\partial \theta_i}\right) - \frac{1}{2}\operatorname{Tr}\left((\boldsymbol{X}^{\mathrm{T}}\boldsymbol{\Sigma_\theta}^{-1}\boldsymbol{X})^{-1}\boldsymbol{X}^{\mathrm{T}}\boldsymbol{\Sigma_\theta}^{-1}\frac{\partial \boldsymbol{\Sigma_\theta}}{\partial \theta_i}\boldsymbol{\Sigma_\theta}^{-1}\boldsymbol{X}\right) \\
&= \frac{1}{2}\boldsymbol{y}^\top\boldsymbol{P_\theta}\frac{\partial \boldsymbol{\Sigma_\theta}}{\partial \theta_i}\boldsymbol{P_\theta}\boldsymbol{y} - \frac{1}{2}\operatorname{Tr}\left(\boldsymbol{\Sigma_\theta}^{-1}\frac{\partial \boldsymbol{\Sigma_\theta}}{\partial \theta_i}\right) - \frac{1}{2}\operatorname{Tr}\left(\boldsymbol{\Sigma_\theta}^{-1}\boldsymbol{X}(\boldsymbol{X}^{\mathrm{T}}\boldsymbol{\Sigma_\theta}^{-1}\boldsymbol{X})^{-1}\boldsymbol{X}^{\mathrm{T}}\boldsymbol{\Sigma_\theta}^{-1}\frac{\partial \boldsymbol{\Sigma_\theta}}{\partial \theta_i}\right) \\
&= \frac{1}{2}\boldsymbol{y}^\top\boldsymbol{P_\theta}\frac{\partial \boldsymbol{\Sigma_\theta}}{\partial \theta_i}\boldsymbol{P_\theta}\boldsymbol{y} - \frac{1}{2}\operatorname{Tr}\left(\left(\boldsymbol{\Sigma_\theta}^{-1} - \boldsymbol{\Sigma_\theta}^{-1}\boldsymbol{X}(\boldsymbol{X}^{\mathrm{T}}\boldsymbol{\Sigma_\theta}^{-1}\boldsymbol{X})^{-1}\boldsymbol{X}^{\mathrm{T}}\boldsymbol{\Sigma_\theta}^{-1}\right)\frac{\partial \boldsymbol{\Sigma_\theta}}{\partial \theta_i}\right) \\
&= \underbrace{\frac{1}{2}\boldsymbol{y}^\top\boldsymbol{P_\theta}\frac{\partial \boldsymbol{\Sigma_\theta}}{\partial \theta_i}\boldsymbol{P_\theta}\boldsymbol{y}}_{\text{squared form}} - \underbrace{\frac{1}{2}\operatorname{Tr}\left(\boldsymbol{P_\theta}\frac{\partial \boldsymbol{\Sigma_\theta}}{\partial \theta_i}\right)}_{\text{trace term}}.
\end{aligned}
$$

Note that this score function is the difference between a quadratic form in the phenotype, $\boldsymbol{y}$, and a trace term that does not involve the phenotype.

Noting that the trace term does not depend on the phenotype data, one can choose to use only the quadratic form in the score function, $\frac{\partial \mathcal{L}(\boldsymbol{\theta})}{\partial \sigma_1^2}$ as the test statistic. For the variance components model considered in this paper (which is additive/independent in the variance components) this quadratic form is equal to $\frac{1}{2}\boldsymbol{y}^\top\boldsymbol{P_\theta}\boldsymbol{K}_1\boldsymbol{P_\theta}\boldsymbol{y}$. We will refer to this as the *score-based test statistic*, although we will leave lemmas and proofs to apply more generally, valid for any of the score components (*i.e.*, for testing any parameter in the mixed model). Note that this form of this test statistic is the same for the single and two kernel cases. However, once we set $\sigma_1^2 = 0$ (the parameter value corresponding to the null hypothesis used to compute the test statistic), then the score-based test statistic can be re-factored in different ways for the one and two kernel cases, as shown in Sections 6 and 6.1.

Next we describe what the distribution of the score-based test statistic is, and how to efficiently compute it. Following that, we describe how to efficiently compute the score-based test statistic itself, for various models.

5.1 Distribution of the score-based test statistic

In the following, we derive the null distribution of the score-based test statistic under the assumption that all variance parameters in the null model are known. In particular, for the matrix $\boldsymbol{\Sigma_\theta}$, it is assumed that the nuisance parameters $\boldsymbol{\theta}$ are known—that is, $\boldsymbol{\Sigma_\theta}$ is assumed to be the true covariance matrix of $\boldsymbol{y}$ under the null distribution. The resulting distribution is a linear combination of χ^2 variables, meaning that we can use Davies method [2] to evaluate significance.

Lemma 2. *Assuming that $\boldsymbol{y} \sim \mathcal{N}(\boldsymbol{X\beta}; \boldsymbol{\Sigma_\theta})$, the score-based test statistic, under the null hypothesis, is distributed as a weighted sum of χ_1^2 variables,*

$$\frac{1}{2}\boldsymbol{y}^\top\boldsymbol{P_\theta}\frac{\partial \boldsymbol{\Sigma_\theta}}{\partial \theta_i}\boldsymbol{P_\theta}\boldsymbol{y} \sim \sum_{n=1}^{N-D}\phi_n\chi_1^2, \tag{10}$$

where the weights, ϕ_n, equal the eigenvalues of the matrix $1/2\boldsymbol{P_\theta}^{\top/2}\frac{\partial \boldsymbol{\Sigma_\theta}}{\partial \theta_i}\boldsymbol{P_\theta}^{1/2}$, and where the matrix $\boldsymbol{P_\theta}^{1/2}$ is any matrix square root of $\boldsymbol{P_\theta}$.

Proof. We start out by re-writing the test statistic, factoring the matrix $\boldsymbol{P_\theta}$ as the product of its square roots ($\boldsymbol{P_\theta} = \boldsymbol{P_\theta}^{1/2}\boldsymbol{P_\theta}^{\top/2}$).

$$\frac{1}{2}\underbrace{\boldsymbol{y}^\top \boldsymbol{P_\theta}^{1/2}}_{\boldsymbol{u}^\top}\boldsymbol{P_\theta}^{\top/2}\frac{\partial \boldsymbol{\Sigma_\theta}}{\partial \theta_i}\boldsymbol{P_\theta}^{1/2}\underbrace{\boldsymbol{P_\theta}^{\top/2}\boldsymbol{y}}_{\boldsymbol{u}}.$$

It is our assumption that under the null distribution $\boldsymbol{y}$ is normal distributed with mean $\boldsymbol{X\beta}$ and covariance $\boldsymbol{\Sigma_\theta}$. As shown in Proposition 20, the $(N-D)$-by-1 vector $\boldsymbol{u} = \boldsymbol{P_\theta}^{\top/2}\boldsymbol{y}$ is normally distributed with zero mean and unit variance.

$$\frac{1}{2}\boldsymbol{u}^\top \boldsymbol{P_\theta}^{T/2}\frac{\partial \boldsymbol{\Sigma_\theta}}{\partial \theta_i}\boldsymbol{P_\theta}^{1/2}\boldsymbol{u}.$$

Now let $\boldsymbol{V\Phi V}^\top$ be the spectral decomposition of $1/2\boldsymbol{P_\theta}^{T/2}\frac{\partial \boldsymbol{\Sigma_\theta}}{\partial \theta_i}\boldsymbol{P_\theta}^{1/2}$, with eigenvalues given by the diagonal of $\boldsymbol{\Phi}$. If we replace this matrix by its spectral decomposition, we obtain the squared form

$$\underbrace{\boldsymbol{u}^\top \boldsymbol{V}}_{\boldsymbol{v}^\top}\boldsymbol{\Phi}\underbrace{\boldsymbol{V}^\top \boldsymbol{u}}_{\boldsymbol{v}},$$

which in turn can be written as a sum weighted by the eigenvalues, ϕ_n,

$$\sum_n \phi_n \cdot v_n^2.$$

Because the matrix of eigenvectors $\boldsymbol{V}$ is orthonormal, it follows that $\boldsymbol{v}$ is also normally distributed with mean zero and unit variance (and therefore each $v_n^2 \sim \chi_1^2$). Therefore, the term $\sum_n \phi_n \cdot v_n^2$ is distributed as the weighted sum of χ_1^2 variables with weights ϕ_n equal to the eigenvalues of $\frac{1}{2}\boldsymbol{P_\theta}^{T/2}\frac{\partial \boldsymbol{\Sigma_\theta}}{\partial \theta_i}\boldsymbol{P_\theta}^{1/2}$.

□

5.1.1 Efficient computation of terms needed for the the score-based test statistic null distribution

To compute a p-value for each set test, we need to compute the null distribution from Lemma 2, which we now show how to do efficiently. In particular, we show how to efficiently compute the eigenvalues of $\frac{1}{2}\boldsymbol{P_\theta}^{T/2}\frac{\partial \boldsymbol{\Sigma_\theta}}{\partial \theta_i}\boldsymbol{P_\theta}^{1/2}$, assuming that $\frac{\partial \boldsymbol{\Sigma_\theta}}{\partial \theta_i}$ factors as $\boldsymbol{GG}^\top$. This assumption is true for our score-based test both with and without the presence of a background kernel. In particular, $\frac{\partial \boldsymbol{\Sigma_\theta}}{\partial \sigma_1^2} = \boldsymbol{K}_1 = \boldsymbol{G}_1\boldsymbol{G}_1^\top$.

Lemma 3. *For the case where $\frac{\partial \boldsymbol{\Sigma_\theta}}{\partial \theta_i}$ factors as $\boldsymbol{GG}^\top$, the non-zero weights of the χ_1^2 distributions in Equation 10 can be computed from the matrix $\frac{1}{2}\boldsymbol{G}^\top\boldsymbol{P_\theta G}$.*

Proof.

$$\frac{1}{2}\boldsymbol{P_\theta}^{T/2}\frac{\partial \boldsymbol{\Sigma_\theta}}{\partial \theta_i}\boldsymbol{P_\theta}^{1/2} = \frac{1}{2}\boldsymbol{P_\theta}^{T/2}\boldsymbol{GG}^\top\boldsymbol{P_\theta}^{1/2}$$

This matrix has the same non-zero eigenvalues as the matrix

$$\frac{1}{2}\boldsymbol{G}^\top \boldsymbol{P}_{\boldsymbol{\theta}}^{1/2}\boldsymbol{P}_{\boldsymbol{\theta}}^{T/2}\boldsymbol{G} = \frac{1}{2}\boldsymbol{G}^\top \boldsymbol{P}_{\boldsymbol{\theta}}\boldsymbol{G}.$$

□

Note that $\frac{1}{2}\boldsymbol{G}^\top \boldsymbol{P}_{\boldsymbol{\theta}}\boldsymbol{G}$ can be efficiently computed by efficiently using the tricks shown in Section 7.3.

6 The single kernel score-based test statistic

Let the covariance $\boldsymbol{\Sigma}_{\boldsymbol{\theta}}$ be defined as $\sigma_{\mathrm{e}}^2\boldsymbol{I} + \sigma_1^2\boldsymbol{K}_1$, with the parameters $\boldsymbol{\theta} = [\sigma_{\mathrm{e}}^2, \sigma_1^2]$ and $\sigma_{\mathrm{e}}^2 > 0$ and $\sigma_1^2 \geq 0$.

We are interested in testing the null hypothesis $\sigma_1^2 = 0$ against the alternative hypothesis $\sigma_1^2 > 0$.

Under the null hypothesis, $\sigma_1^2 = 0$, the matrix $\boldsymbol{P}_{\boldsymbol{\theta}}$ reduces to $\sigma_{\mathrm{e}}^{-2}\boldsymbol{S}$. It follows that the score with respect to the parameter σ_1^2 is

$$\begin{aligned}\frac{\partial \mathcal{L}\left(\sigma_{\mathrm{e}}^2, \sigma_1^2 = 0\right)}{\partial \sigma_1^2} &= \frac{1}{2}\boldsymbol{y}^\top \boldsymbol{P}_{\boldsymbol{\theta}}\frac{\partial \boldsymbol{\Sigma}_{\boldsymbol{\theta}}}{\partial \sigma_1^2}\boldsymbol{P}_{\boldsymbol{\theta}}\boldsymbol{y} - \frac{1}{2}\operatorname{Tr}\left(\boldsymbol{P}_{\boldsymbol{\theta}}\frac{\partial \boldsymbol{\Sigma}_{\boldsymbol{\theta}}}{\partial \sigma_1^2}\right) \\ &= \underbrace{\frac{1}{2\sigma_{\mathrm{e}}^4}\boldsymbol{y}^\top \boldsymbol{S}\boldsymbol{K}_1\boldsymbol{S}\boldsymbol{y}}_{\text{squared form}} - \underbrace{\frac{1}{2\sigma_{\mathrm{e}}^2}\operatorname{Tr}\left(\boldsymbol{S}\boldsymbol{K}_1\right)}_{\text{trace term}}.\end{aligned} \tag{11}$$

6.0.2 Low rank single kernel score-based test statistic

Let the matrix $\boldsymbol{K}_1$ be defined as $\boldsymbol{G}_1\boldsymbol{G}_1^\top$, where $\boldsymbol{G}_1$ is of dimension $N \times k$) contains the set of SNPs being tested. Then, the single kernel score with respect to the parameter σ_1^2 can be evaluated as

$$\begin{aligned}\frac{\partial \mathcal{L}\left(\sigma_{\mathrm{e}}^2, \sigma_1^2 = 0\right)}{\partial \sigma_1^2} &= \frac{1}{2\sigma_{\mathrm{e}}^4}\boldsymbol{y}^\top \boldsymbol{S}\boldsymbol{K}_1\boldsymbol{S}\boldsymbol{y} - \frac{1}{2\sigma_{\mathrm{e}}^2}\operatorname{Tr}\left(\boldsymbol{S}\boldsymbol{K}_1\right) \\ &= \frac{1}{2\sigma_{\mathrm{e}}^4}\boldsymbol{y}^\top \boldsymbol{S}\boldsymbol{G}_1\boldsymbol{G}_1^\top\boldsymbol{S}\boldsymbol{y} - \frac{1}{2\sigma_{\mathrm{e}}^2}\operatorname{Tr}\left(\boldsymbol{S}\boldsymbol{G}_1\boldsymbol{G}_1^\top\right) \\ &= \underbrace{\frac{1}{2\sigma_{\mathrm{e}}^4}(\boldsymbol{y}^\top \boldsymbol{S}\boldsymbol{G}_1)(\boldsymbol{G}_1^\top\boldsymbol{S}\boldsymbol{y})}_{\text{squared form}} - \underbrace{\frac{1}{2\sigma_{\mathrm{e}}^2}\operatorname{Tr}\left(\boldsymbol{G}_1^\top\boldsymbol{S}\boldsymbol{G}_1\right)}_{\text{trace term}}.\end{aligned} \tag{12}$$

6.1 The two-kernel score-based test statistic

Let the covariance $\boldsymbol{\Sigma}_{\boldsymbol{\theta}}$ be defined as $\sigma_{\mathrm{e}}^2\boldsymbol{I} + \sigma_{\mathrm{g}}^2\boldsymbol{K}_{\mathrm{g}} + \sigma_1^2\boldsymbol{K}$, with parameters $\boldsymbol{\theta} = [\sigma_{\mathrm{e}}^2, \sigma_{\mathrm{g}}^2, \sigma_1^2]$ and $\sigma_{\mathrm{e}}^2 > 0$, $\sigma_{\mathrm{g}}^2 \geq 0$, $\sigma_1^2 \geq 0$.

We are interested in testing the null hypothesis $\sigma_1^2 = 0$, vs. the alternative hypothesis $\sigma_1^2 > 0$.

The score with respect to the parameter σ_1^2 is

$$\begin{aligned}\frac{\partial \mathcal{L}\left(\sigma_{\mathrm{e}}^2, \sigma_{\mathrm{g}}^2, \sigma_1^2=0\right)}{\partial \sigma_1^2} &= \frac{1}{2}\boldsymbol{y}^\top \boldsymbol{P}_{\boldsymbol{\theta}} \frac{\partial \boldsymbol{\Sigma}_{\boldsymbol{\theta}}}{\partial \sigma_1^2} \boldsymbol{P}_{\boldsymbol{\theta}} \boldsymbol{y} - \frac{1}{2} \operatorname{Tr}\left(\boldsymbol{P}_{\boldsymbol{\theta}} \frac{\partial \boldsymbol{\Sigma}_{\boldsymbol{\theta}}}{\partial \sigma_1^2}\right) \\ &= \underbrace{\frac{1}{2}\boldsymbol{y}^\top \boldsymbol{P}_{\boldsymbol{\theta}} \boldsymbol{K}_1 \boldsymbol{P}_{\boldsymbol{\theta}} \boldsymbol{y}}_{\text{squared form}} - \underbrace{\frac{1}{2} \operatorname{Tr}\left(\boldsymbol{P}_{\boldsymbol{\theta}} \boldsymbol{K}_1\right)}_{\text{trace term}}\end{aligned} \tag{13}$$

6.1.1 Low rank two kernel score-based test statistic

Let the matrix $\boldsymbol{K}_1$ be defined as $\boldsymbol{G}_1\boldsymbol{G}_1^\top$. Further, let the matrix $\boldsymbol{K}_g = \boldsymbol{G}_g\boldsymbol{G}_g^\top$ be low rank. Then, the single kernel score with respect to the parameter σ_1^2 can be evaluated as

$$\begin{aligned}\frac{\partial \mathcal{L}\left(\sigma_{\mathrm{e}}^2, \sigma_{\mathrm{g}}^2, \sigma_1^2=0\right)}{\partial \sigma_1^2} &= \frac{1}{2}\boldsymbol{y}^\top \boldsymbol{P}_{\boldsymbol{\theta}} \boldsymbol{G}_1 \boldsymbol{G}_1^\top \boldsymbol{P}_{\boldsymbol{\theta}} \boldsymbol{y} - \frac{1}{2} \operatorname{Tr}\left(\boldsymbol{P}_{\boldsymbol{\theta}} \boldsymbol{G}_1 \boldsymbol{G}_1^\top\right) \\ &= \underbrace{\frac{1}{2}(\boldsymbol{y}^\top \boldsymbol{P}_{\boldsymbol{\theta}} \boldsymbol{G}_1)(\boldsymbol{G}_1^\top \boldsymbol{P}_{\boldsymbol{\theta}} \boldsymbol{y})}_{\text{squared form}} - \underbrace{\frac{1}{2} \operatorname{Tr}\left(\boldsymbol{G}_1^\top \boldsymbol{P}_{\boldsymbol{\theta}} \boldsymbol{G}_1\right)}_{\text{trace term}}.\end{aligned} \tag{14}$$

Using Lemma 11 and Proposition 9, we can efficiently evaluate the squared form without computing $\boldsymbol{P}_{\boldsymbol{\theta}}$, and do so in a manner which is linear in N.

7 Additional derivations

The following derivations and proofs have been adapted from [1]. Please see the preamble of this document for variable definitions when no reminder is provided.

Recall that $\boldsymbol{S} = \left(\boldsymbol{I}_N - \boldsymbol{X}\left(\boldsymbol{X}^\top\boldsymbol{X}\right)^{-1}\boldsymbol{X}^\top\right)$ and has rank $N-D$, and that $\boldsymbol{P}_{\boldsymbol{\theta}} = \left(\boldsymbol{\Sigma}_{\boldsymbol{\theta}}^{-1} - \boldsymbol{\Sigma}_{\boldsymbol{\theta}}^{-1}\boldsymbol{X}(\boldsymbol{X}^\mathrm{T}\boldsymbol{\Sigma}_{\boldsymbol{\theta}}^{-1}\boldsymbol{X})^{-1}\boldsymbol{X}^\top\boldsymbol{\Sigma}_{\boldsymbol{\theta}}^{-1}\right)$ is an N-by-N symmetric matrix with has rank $N-D$.

Proposition 4. $\boldsymbol{SX} = \boldsymbol{0}$.

Proof.

$$\begin{aligned}\boldsymbol{SX} &= \boldsymbol{IX} - \boldsymbol{X}\underbrace{\left(\boldsymbol{X}^\top\boldsymbol{X}\right)^{-1}\boldsymbol{X}^\top\boldsymbol{X}}_{\boldsymbol{I}} \\ &= \boldsymbol{X} - \boldsymbol{X} \\ &= \boldsymbol{0}.\end{aligned}$$

□

Proposition 5. $\boldsymbol{SS} = \boldsymbol{S}$.

Proof.

$$\begin{aligned}\boldsymbol{SS} &= \boldsymbol{SI} - \underbrace{\boldsymbol{SX}}_{\boldsymbol{0}}\left(\boldsymbol{X}^\top\boldsymbol{X}\right)^{-1}\boldsymbol{X}^\top \\ &= \boldsymbol{S}.\end{aligned}$$

□

Proposition 6. $\boldsymbol{P_\theta X} = \mathbf{0}$.

Proof.

$$\begin{aligned}\boldsymbol{P_\theta X} &= \boldsymbol{\Sigma}_{\boldsymbol{\theta}}^{-1}\boldsymbol{X} - \boldsymbol{\Sigma}_{\boldsymbol{\theta}}^{-1}\boldsymbol{X}\underbrace{\left(\boldsymbol{X}^\top\boldsymbol{\Sigma}_{\boldsymbol{\theta}}^{-1}\boldsymbol{X}\right)^{-1}\boldsymbol{X}^\top\boldsymbol{\Sigma}_{\boldsymbol{\theta}}^{-1}\boldsymbol{X}}_{\boldsymbol{I}} \\ &= \boldsymbol{X}\boldsymbol{\Sigma}_{\boldsymbol{\theta}}^{-1} - \boldsymbol{X}\boldsymbol{\Sigma}_{\boldsymbol{\theta}}^{-1} \\ &= \mathbf{0}.\end{aligned}$$

□

Proposition 7. $\boldsymbol{P_\theta S} = \boldsymbol{P_\theta}$.

Proof.

$$\begin{aligned}\boldsymbol{P_\theta S} &= \boldsymbol{P_\theta} - \underbrace{\boldsymbol{P_\theta X}}_{\mathbf{0}}\left(\boldsymbol{X}^\top\boldsymbol{X}\right)^{-1}\boldsymbol{X}^\top \\ &= \boldsymbol{P_\theta}.\end{aligned}$$

□

7.1 Matrix derivative of $\boldsymbol{P_\theta}$

Proposition 8. $\frac{\partial \boldsymbol{P_\theta}}{\partial\theta_i} = -\boldsymbol{P_\theta}\frac{\partial\boldsymbol{\Sigma_\theta}}{\partial\theta_i}\boldsymbol{P_\theta}$.

Proof.

$$\begin{aligned}\frac{\partial \boldsymbol{P_\theta}}{\partial\theta_i} =& -\boldsymbol{\Sigma}_{\boldsymbol{\theta}}^{-1}\frac{\partial\boldsymbol{\Sigma_\theta}}{\partial\theta_i}\boldsymbol{\Sigma}_{\boldsymbol{\theta}}^{-1} + \boldsymbol{\Sigma}_{\boldsymbol{\theta}}^{-1}\frac{\partial\boldsymbol{\Sigma_\theta}}{\partial\theta_i}\boldsymbol{\Sigma}_{\boldsymbol{\theta}}^{-1}\boldsymbol{X}(\boldsymbol{X}^{\mathrm{T}}\boldsymbol{\Sigma}_{\boldsymbol{\theta}}^{-1}\boldsymbol{X})^{-1}\boldsymbol{X}^{\mathrm{T}}\boldsymbol{\Sigma}_{\boldsymbol{\theta}}^{-1} + \boldsymbol{\Sigma}_{\boldsymbol{\theta}}^{-1}\boldsymbol{X}(\boldsymbol{X}^{\mathrm{T}}\boldsymbol{\Sigma}_{\boldsymbol{\theta}}^{-1}\boldsymbol{X})^{-1}\boldsymbol{X}^{\mathrm{T}}\boldsymbol{\Sigma}_{\boldsymbol{\theta}}^{-1}\frac{\partial\boldsymbol{\Sigma_\theta}}{\partial\theta_i}\boldsymbol{\Sigma}_{\boldsymbol{\theta}}^{-1} \\ & - \boldsymbol{\Sigma}_{\boldsymbol{\theta}}^{-1}\boldsymbol{X}(\boldsymbol{X}^{\mathrm{T}}\boldsymbol{\Sigma}_{\boldsymbol{\theta}}^{-1}\boldsymbol{X})^{-1}\boldsymbol{X}^{\mathrm{T}}\boldsymbol{\Sigma}_{\boldsymbol{\theta}}^{-1}\frac{\partial\boldsymbol{\Sigma_\theta}}{\partial\theta_i}\boldsymbol{\Sigma}_{\boldsymbol{\theta}}^{-1}\boldsymbol{X}(\boldsymbol{X}^{\mathrm{T}}\boldsymbol{\Sigma}_{\boldsymbol{\theta}}^{-1}\boldsymbol{X})^{-1}\boldsymbol{X}^{\mathrm{T}}\boldsymbol{\Sigma}_{\boldsymbol{\theta}}^{-1} \\ =& -\left(\boldsymbol{\Sigma}_{\boldsymbol{\theta}}^{-1} - \boldsymbol{\Sigma}_{\boldsymbol{\theta}}^{-1}\boldsymbol{X}(\boldsymbol{X}^{\mathrm{T}}\boldsymbol{\Sigma}_{\boldsymbol{\theta}}^{-1}\boldsymbol{X})^{-1}\boldsymbol{X}^{\mathrm{T}}\boldsymbol{\Sigma}_{\boldsymbol{\theta}}^{-1}\right)\frac{\partial\boldsymbol{\Sigma_\theta}}{\partial\theta_i}\left(\boldsymbol{\Sigma}_{\boldsymbol{\theta}}^{-1} - \boldsymbol{\Sigma}_{\boldsymbol{\theta}}^{-1}\boldsymbol{X}(\boldsymbol{X}^{\mathrm{T}}\boldsymbol{\Sigma}_{\boldsymbol{\theta}}^{-1}\boldsymbol{X})^{-1}\boldsymbol{X}^{\mathrm{T}}\boldsymbol{\Sigma}_{\boldsymbol{\theta}}^{-1}\right) \\ =& -\boldsymbol{P_\theta}\frac{\partial\boldsymbol{\Sigma_\theta}}{\partial\theta_i}\boldsymbol{P_\theta}.\end{aligned}$$

□

7.2 Efficient multiplication with $\boldsymbol{S}$

Proposition 9. *For any N-by-1-vector $\boldsymbol{a}$ multiplication with $\boldsymbol{S}$ can be done efficiently in time complexity $O(ND)$, without explicitly computing $\boldsymbol{S}$.*

Proof.

$$\begin{aligned}\boldsymbol{S}\boldsymbol{a} &= \left(\boldsymbol{I} - \boldsymbol{X}\left(\boldsymbol{X}^\top\boldsymbol{X}\right)\boldsymbol{X}^\top\right)\boldsymbol{a}\\ &= \boldsymbol{a} - \boldsymbol{X}\underbrace{\left(\boldsymbol{X}^\top\boldsymbol{X}\right)\boldsymbol{X}^\top}_{\boldsymbol{X}^\dagger}\boldsymbol{a}\\ &= \boldsymbol{a} - \boldsymbol{X}\underbrace{\left(\boldsymbol{X}^\dagger\boldsymbol{a}\right)}_{\text{OLS}}.\end{aligned}$$

□

The resulting term is nothing but the ordinary least squares (OLS) regression residuals after regressing out $\boldsymbol{X}$. Note, that the pseudoinverse of the covariates, $\boldsymbol{X}^\dagger$, need only be computed once (in $O(ND^2)$) and then can be re-used across different $\boldsymbol{a}$.For multiplication of matrices by $\boldsymbol{S}$ the result is applied by treating each row or column as a vector in the multiplication.

7.3 Spectral decomposition of $\boldsymbol{P_\theta}$ and efficient computation of squared forms in $\boldsymbol{P_\theta}$

One computational bottleneck for the score-based test is computation of a matrix square root of $\boldsymbol{P_\theta}$ ($\boldsymbol{P_\theta}^{1/2}$), which can be obtained directly from the spectral decomposition of $\boldsymbol{P_\theta}$. Therefore, we seek to efficiently compute $\boldsymbol{P_\theta}^{1/2}$. To do so, we note that $\sigma_{\mathrm{g}}^2\boldsymbol{P_\theta} = \left(\boldsymbol{S}\left(\boldsymbol{K}_{\mathrm{g}} + \delta\boldsymbol{I}_N\right)\boldsymbol{S}\right)^\dagger$, which can be seen using Lemma 12. Below we show how to compute the a matrix square root of $\left(\boldsymbol{S}\left(\boldsymbol{K}_{\mathrm{g}} + \delta\boldsymbol{I}_N\right)\boldsymbol{S}\right)^\dagger$.

The following lemma was stated without proof in [7] and is proved in Proposition C.15 of [1]:

Lemma 10. *Let the economy spectral decomposition of $\boldsymbol{S}\left(\boldsymbol{K}_{\mathrm{g}} + \boldsymbol{I}_N\right)\boldsymbol{S}$ be $\boldsymbol{U}\left(\boldsymbol{\Lambda} + \boldsymbol{I}_{N-D}\right)\boldsymbol{U}^\top$. Then the economy spectral decomposition of $\boldsymbol{S}\left(\boldsymbol{K}_{\mathrm{g}} + \delta\boldsymbol{I}_N\right)\boldsymbol{S}$ is given by $\boldsymbol{U}\left(\boldsymbol{\Lambda} + \delta\boldsymbol{I}_{N-D}\right)\boldsymbol{U}^\top$, where $\left(\boldsymbol{\Lambda} + \delta\boldsymbol{I}_{N-D}\right)$ is a diagonal matrix holding the $N-D$ non-zero eigenvalues of $\boldsymbol{S}\left(\boldsymbol{K} + \delta\boldsymbol{I}_N\right)\boldsymbol{S}$ (in contrast, the first $N-D$ eigenvectors remain unchanged and are given as columns of $\boldsymbol{U}$.)*

Proof.

$$\begin{aligned}\boldsymbol{S}\left(\boldsymbol{K}_{\mathrm{g}} + \delta\boldsymbol{I}_N\right)\boldsymbol{S} =& \boldsymbol{S}\left(\left(\boldsymbol{K}_{\mathrm{g}} + \boldsymbol{I}_N\right) + (\delta - 1)\boldsymbol{I}_N\right)\boldsymbol{S}\\ =& \boldsymbol{S}\left(\boldsymbol{K}_{\mathrm{g}} + \boldsymbol{I}\right)\boldsymbol{S} + (\delta-1)\underbrace{\boldsymbol{S}\boldsymbol{I}_N\boldsymbol{S}}_{\boldsymbol{S}}\\ =& \boldsymbol{U}\left(\boldsymbol{\Lambda} + \boldsymbol{I}\right)\boldsymbol{U}^\top + (\delta-1)\boldsymbol{U}\boldsymbol{I}_{N-D}\boldsymbol{U}^\top\\ =& \boldsymbol{U}\left(\boldsymbol{\Lambda} + \delta\boldsymbol{I}_{N-D}\right)\boldsymbol{U}^\top,\end{aligned}$$

where we used idempotency of $\boldsymbol{S}$ and Lemma 17 to replace $\boldsymbol{S}$ by $\boldsymbol{U}\boldsymbol{U}^\top$.

The proof relies on $\boldsymbol{K}_{\mathrm{g}} + \delta\boldsymbol{I}$ to be full rank, which is always true for $\delta > 0$. □

Under certain conditions ("low rank"), these computations can be made more efficient. In particular, for the case, where $\boldsymbol{K}_{\mathrm{g}}$ is factored as $\boldsymbol{K} = \boldsymbol{G}_{\mathrm{g}}\boldsymbol{G}_{\mathrm{g}}^\top$, with the N-by-k_{g} factor $\boldsymbol{G}_{\mathrm{g}}$, only the singular value decomposition of the N-by-k_{g} matrix $\boldsymbol{S}\boldsymbol{G}_{\mathrm{g}}$ has to be computed (which is linear in N, rather than cubic as taking the singular value decomposition of $\boldsymbol{K}_{\mathrm{g}}$ would be). This can be seen by noting that $\boldsymbol{S}\boldsymbol{G}_{\mathrm{g}}$ is a matrix square root of $\boldsymbol{S}\boldsymbol{K}_{\mathrm{g}}\boldsymbol{S}$, and therefore that the economy SVD $\boldsymbol{U}\boldsymbol{\Lambda}^{1/2}\boldsymbol{V}^\top$ of $\boldsymbol{S}\boldsymbol{G}_{\mathrm{g}}$ gives the economy eigenvalue decomposition of $\boldsymbol{S}\boldsymbol{K}_{\mathrm{g}}\boldsymbol{S}$, which is given by $\boldsymbol{U}\boldsymbol{\Lambda}\boldsymbol{U}^\top$.

Let $\boldsymbol{U}_\perp$ be an N-by-$(N-k_{\mathrm{g}}-D)$ matrix with orthogonal columns that are all orthogonal to the columns of both $\boldsymbol{U}$, as well as $\boldsymbol{X}$. Then the economy eigenvalue decomposition of $\boldsymbol{S}\left(\boldsymbol{K}_{\mathrm{g}}+\delta\boldsymbol{I}_N\right)\boldsymbol{S}$ is given by

$$\boldsymbol{S}\left(\boldsymbol{K}_{\mathrm{g}}+\delta\boldsymbol{I}_N\right)\boldsymbol{S}=\left[\begin{array}{cc}\boldsymbol{U} & \boldsymbol{U}_\perp\end{array}\right]\left[\begin{array}{cc}\boldsymbol{\Lambda}+\delta\boldsymbol{I}_{k_{\mathrm{g}}} & \mathbf{0}\\ \mathbf{0} & \delta\boldsymbol{I}_{N-D-k_{\mathrm{g}}}\end{array}\right]\left[\begin{array}{cc}\boldsymbol{U} & \boldsymbol{U}_\perp\end{array}\right]^\top.$$

Then the economy eigenvalue decomposition of $\boldsymbol{P_\theta}$ can be obtained as follows:

$$\sigma_{\mathrm{g}}^2\boldsymbol{P_\theta}=\left(\boldsymbol{S}\left(\boldsymbol{K}_{\mathrm{g}}+\delta\boldsymbol{I}_N\right)\boldsymbol{S}\right)^\dagger$$

using Lemma 12

$$=\left[\begin{array}{cc}\boldsymbol{U} & \boldsymbol{U}_\perp\end{array}\right]\left(\left[\begin{array}{cc}\boldsymbol{\Lambda} & \mathbf{0}\\ \mathbf{0} & \mathbf{0}\end{array}\right]+\delta\boldsymbol{I}_{N-D}\right)^{-1}\left[\begin{array}{cc}\boldsymbol{U} & \boldsymbol{U}_\perp\end{array}\right]^\top,$$

because $\boldsymbol{K}=\boldsymbol{G}_{\mathrm{g}}\boldsymbol{G}_{\mathrm{g}}^\top$ is low rank,

$$\begin{aligned}
&=\left[\begin{array}{cc}\boldsymbol{U} & \boldsymbol{U}_\perp\end{array}\right]\left[\begin{array}{cc}\boldsymbol{\Lambda}+\delta\boldsymbol{I}_{k_{\mathrm{g}}} & \mathbf{0}\\ \mathbf{0} & \delta\boldsymbol{I}_{N-D-k_{\mathrm{g}}}\end{array}\right]^{-1}\left[\begin{array}{cc}\boldsymbol{U} & \boldsymbol{U}_\perp\end{array}\right]^\top\\
&=\boldsymbol{U}\left(\boldsymbol{\Lambda}+\delta\boldsymbol{I}_{k_{\mathrm{g}}}\right)^{-1}\boldsymbol{U}^\top+\frac{1}{\delta}\boldsymbol{U}_\perp\boldsymbol{I}_{N-D-k_{\mathrm{g}}}^{-1}\boldsymbol{U}_\perp^\top\\
&=\boldsymbol{U}\left(\boldsymbol{\Lambda}+\delta\boldsymbol{I}_{k_{\mathrm{g}}}\right)^{-1}\boldsymbol{U}^\top+\frac{1}{\delta}\boldsymbol{U}_\perp\boldsymbol{U}_\perp^\top+\underbrace{\frac{1}{\delta}\left(\boldsymbol{U}\boldsymbol{U}^\top-\boldsymbol{U}\boldsymbol{U}^\top\right)}_{\mathbf{0}}\\
&=\boldsymbol{U}\left(\boldsymbol{\Lambda}+\delta\boldsymbol{I}_{k_{\mathrm{g}}}\right)^{-1}\boldsymbol{U}^\top+\frac{1}{\delta}\underbrace{\left(\boldsymbol{U}_\perp\boldsymbol{U}_\perp^\top+\boldsymbol{U}\boldsymbol{U}^\top\right)}_{\left[\begin{array}{cc}\boldsymbol{U} & \boldsymbol{U}_\perp\end{array}\right]\left[\begin{array}{cc}\boldsymbol{U} & \boldsymbol{U}_\perp\end{array}\right]^\top=\boldsymbol{S}}-\frac{1}{\delta}\boldsymbol{U}\boldsymbol{U}^\top\\
&=\boldsymbol{U}\left(\boldsymbol{\Lambda}+\delta\boldsymbol{I}_{k_{\mathrm{g}}}\right)^{-1}\boldsymbol{U}^\top+\frac{1}{\delta}\left(\boldsymbol{S}-\boldsymbol{U}\boldsymbol{U}^\top\right).
\end{aligned}\tag{15}$$

Lemma 11. *It follows that when $\boldsymbol{K}_{\mathrm{g}}$ is factored as $\boldsymbol{K}_{\mathrm{g}}=\boldsymbol{G}_{\mathrm{g}}\boldsymbol{G}_{\mathrm{g}}^\top$, then for all $\boldsymbol{a}$ and $\boldsymbol{b}$, any squared form in $\boldsymbol{P_\theta}$, written here as $\boldsymbol{a}^\top\boldsymbol{P_\theta}\boldsymbol{b}$ can be computed efficiently only using the singular value decomposition of $\boldsymbol{S}\boldsymbol{G}_{\mathrm{g}}$.*

Proof.

$$\begin{aligned}
\boldsymbol{a}^\top\boldsymbol{P_\theta}\boldsymbol{b}=&\boldsymbol{a}^\top\boldsymbol{S}\boldsymbol{P_\theta}\boldsymbol{S}\boldsymbol{b}\\
=&\frac{1}{\sigma_{\mathrm{g}}^2}\boldsymbol{a}^\top\boldsymbol{S}\left(\boldsymbol{U}\left(\boldsymbol{\Lambda}+\delta\boldsymbol{I}_{k_{\mathrm{g}}}\right)^{-1}\boldsymbol{U}^\top+\frac{1}{\delta}\left(\boldsymbol{S}-\boldsymbol{U}\boldsymbol{U}^\top\right)\right)\boldsymbol{S}\boldsymbol{b}\\
=&\frac{1}{\sigma_{\mathrm{g}}^2}\boldsymbol{a}^\top\boldsymbol{S}\boldsymbol{U}\left(\boldsymbol{\Lambda}+\delta\boldsymbol{I}_{k_{\mathrm{g}}}\right)^{-1}\boldsymbol{U}^\top\boldsymbol{S}\boldsymbol{b}+\frac{1}{\delta}\boldsymbol{a}^\top\underbrace{\boldsymbol{S}\boldsymbol{S}\boldsymbol{S}}_{\boldsymbol{S}}\boldsymbol{b}-\frac{1}{\delta}\boldsymbol{a}^\top\boldsymbol{S}\boldsymbol{U}\boldsymbol{U}^\top\boldsymbol{S}\boldsymbol{b}\\
=&\frac{1}{\sigma_{\mathrm{g}}^2}\boldsymbol{a}^\top\boldsymbol{S}\boldsymbol{U}\left(\boldsymbol{\Lambda}+\delta\boldsymbol{I}_{k_{\mathrm{g}}}\right)^{-1}\boldsymbol{U}^\top\boldsymbol{S}\boldsymbol{b}+\frac{1}{\delta}\boldsymbol{a}^\top\boldsymbol{S}\boldsymbol{b}-\frac{1}{\delta}\boldsymbol{a}^\top\boldsymbol{S}\boldsymbol{U}\boldsymbol{U}^\top\boldsymbol{S}\boldsymbol{b}.
\end{aligned}$$

□

7.4 Moore-Penrose pseudoinverse of $\boldsymbol{P_\theta}$

Lemma 12. *$\boldsymbol{S\Sigma_\theta S}$ is the Moore-Penrose pseudoinverse of $\boldsymbol{P_\theta}$ [1].*

Proof. The four properties of the Moore-Penrose pseudoinverse are proven below in Propositions 13 to 16, thereby completing the proof. □

This Lemma was stated and used in [7]. A proof can be found in Lemma C.10 of [1].

Proposition 13. *$(\boldsymbol{S\Sigma_\theta S})\,\boldsymbol{P_\theta}$ is symmetric.*

Proof.

$$\begin{aligned}
(\boldsymbol{S\Sigma_\theta S})\,\boldsymbol{P_\theta} =& \boldsymbol{S\Sigma_\theta}\underbrace{\boldsymbol{SP_\theta}}_{\boldsymbol{P_\theta}} \\
=& \boldsymbol{S\Sigma_\theta P_\theta} \\
=& \boldsymbol{S}\underbrace{\boldsymbol{\Sigma_\theta\Sigma_\theta^{-1}}}_{\boldsymbol{I}} - \boldsymbol{S}\underbrace{\boldsymbol{\Sigma_\theta\Sigma_\theta^{-1}}}_{\boldsymbol{I}}\boldsymbol{X}\left(\boldsymbol{X}^\top\boldsymbol{\Sigma_\theta^{-1}X}\right)^{-1}\boldsymbol{X}^\top\boldsymbol{\Sigma_\theta^{-1}} \\
=& \boldsymbol{S} - \underbrace{\boldsymbol{SX}}_{\boldsymbol{0}}\left(\boldsymbol{X}^\top\boldsymbol{\Sigma_\theta^{-1}X}\right)^{-1}\boldsymbol{X}^\top\boldsymbol{\Sigma_\theta^{-1}} \\
=& \boldsymbol{S}.
\end{aligned}$$

As $\boldsymbol{S}$ is symmetric, $(\boldsymbol{S\Sigma_\theta S})\,\boldsymbol{P_\theta}$ is also symmetric. □

Proposition 14. *$\boldsymbol{P_\theta}\,(\boldsymbol{S\Sigma_\theta S})$ is symmetric.*

Proof.

$$\boldsymbol{P_\theta}\,(\boldsymbol{S\Sigma_\theta S}) = \boldsymbol{S}.$$

As $\boldsymbol{S}$ is symmetric, $\boldsymbol{P_\theta}\,(\boldsymbol{S\Sigma_\theta S})$ is also symmetric. □

Proposition 15. *$\boldsymbol{P_\theta}$ is a weak inverse of $\boldsymbol{S\Sigma_\theta S}$.*

Proof.

$$\begin{aligned}
\underbrace{\boldsymbol{P_\theta}\,(\boldsymbol{S\Sigma_\theta S})}_{\boldsymbol{S}}\,\boldsymbol{P_\theta} =& \boldsymbol{SP_\theta} \\
=& \boldsymbol{P_\theta}.
\end{aligned}$$

□

Proposition 16. *$\boldsymbol{S\Sigma_\theta S}$ is a weak inverse of $\boldsymbol{P_\theta}$.*

Proof.

$$\begin{aligned}
\underbrace{(\boldsymbol{S\Sigma_\theta S})\,\boldsymbol{P_\theta}}_{\boldsymbol{S}}\,(\boldsymbol{S\Sigma_\theta S}) =& \underbrace{\boldsymbol{SS}}_{\boldsymbol{S}}\,\boldsymbol{\Sigma_\theta S} \\
=& \boldsymbol{S\Sigma_\theta S}.
\end{aligned}$$

□

Lemma 17. *Let $\boldsymbol{U\Lambda U}^\top$ be the* **economy** *spectral decomposition of $(\boldsymbol{S\Sigma_\theta S})$, where $\boldsymbol{\Lambda}$ is an $(N-D)$-by-$(N-D)$ diagonal matrix, holding the non-zero eigenvalues of $(\boldsymbol{S\Sigma_\theta S})$, and $\boldsymbol{U}$ is the N-by-$(N-D)$ matrix, holding the corresponding $N-D$ eigenvectors of $(\boldsymbol{S\Sigma_\theta S})$ as columns. Then $\boldsymbol{S} = \boldsymbol{UU}^\top$ [7].*

Proof.

$$\begin{aligned}
\boldsymbol{S} &= (\boldsymbol{S\Sigma_\theta S})\,\boldsymbol{P_\theta} \\
&= (\boldsymbol{S\Sigma_\theta S})\,(\boldsymbol{S\Sigma_\theta S})^\dagger \\
&= \left(\boldsymbol{U\Lambda U}^\top\right)\left(\boldsymbol{U\Lambda}^{-1}\boldsymbol{U}^\top\right) \\
&= \boldsymbol{U\Lambda}\underbrace{\boldsymbol{U}^\top\boldsymbol{U}}_{\boldsymbol{I}}\boldsymbol{\Lambda}^{-1}\boldsymbol{U}^\top \\
&= \boldsymbol{U}\underbrace{\boldsymbol{\Lambda\Lambda}^{-1}}_{\boldsymbol{I}}\boldsymbol{U}^\top \\
&= \boldsymbol{UU}^\top.
\end{aligned}$$

□

Proposition 18. *Let $\boldsymbol{U\Lambda U}^\top$ be the* **economy** *spectral decomposition of $(\boldsymbol{S\Sigma_\theta S})$, where $\boldsymbol{\Lambda}$ is an $(N-D)$-by-$(N-D)$ diagonal matrix, holding the non-zero eigenvalues of $(\boldsymbol{S\Sigma_\theta S})$, and $\boldsymbol{U}$ is the N-by-$(N-D)$ matrix, holding the corresponding $N-D$ eigenvectors of $(\boldsymbol{S\Sigma_\theta S})$ as columns. Then $\boldsymbol{U}^\top\boldsymbol{\Sigma_\theta U} = \boldsymbol{\Lambda}$ [7].*

Proof.

$$\begin{aligned}
\boldsymbol{U}^\top\boldsymbol{\Sigma_\theta U} &= \overbrace{\boldsymbol{U}^\top\boldsymbol{U}}^{\boldsymbol{I}}\boldsymbol{U}^\top\boldsymbol{\Sigma_\theta U}\overbrace{\boldsymbol{U}^\top\boldsymbol{U}}^{\boldsymbol{I}} \\
&= \boldsymbol{U}^\top\boldsymbol{S\Sigma_\theta S U} \\
&= \underbrace{\boldsymbol{U}^\top\boldsymbol{U}}_{\boldsymbol{I}}\boldsymbol{\Lambda}\underbrace{\boldsymbol{U}^\top\boldsymbol{U}}_{\boldsymbol{I}} \\
&= \boldsymbol{\Lambda}.
\end{aligned}$$

□

7.5 Pseudo-determinant of $\boldsymbol{P_\theta}$

Proposition 19.

$$\frac{1}{2}\log|\boldsymbol{P_\theta}|_+ = -\frac{1}{2}\log|\boldsymbol{\Sigma_\theta}| + \frac{1}{2}\log|\boldsymbol{X}^\mathrm{T}\boldsymbol{X}| - \frac{1}{2}\log|\boldsymbol{X}^\mathrm{T}\boldsymbol{\Sigma}_{\boldsymbol{\theta}}^{-1}\boldsymbol{X}|$$

This Proposition was stated without a proof in [3]. A proof was provided in Proposition C.7 in [1].

Proof. From Lemma 12 we know that $\boldsymbol{P_\theta} = (\boldsymbol{S\Sigma_\theta S})^\dagger$.

$$|\boldsymbol{P_\theta}|_+ = |\boldsymbol{S\Sigma_\theta S}|_+^{-1}$$

Let $\boldsymbol{U\Lambda U}^\top$ be the **economy** spectral decomposition of $(\boldsymbol{S\Sigma_\theta S})$, where $\boldsymbol{\Lambda}$ is an $(N-D)$-by-$(N-D)$ diagonal matrix, holding the non-zero eigenvalues of $(\boldsymbol{S\Sigma_\theta S})$, and $\boldsymbol{U}$ is the N-by-$(N-D)$ matrix, holding the corresponding $N-D$ eigenvectors of $(\boldsymbol{S\Sigma_\theta S})$ as columns.

$$
\begin{aligned}
|\boldsymbol{P_\theta}|_+ =& |\boldsymbol{S\Sigma_\theta S}|_+^{-1} \\
=& |\boldsymbol{\Lambda}|^{-1} \cdot \overbrace{|\boldsymbol{X}^\top\boldsymbol{\Sigma}_\theta^{-1}\boldsymbol{X}| \cdot |\boldsymbol{X}^\top\boldsymbol{\Sigma}_\theta^{-1}\boldsymbol{X}|^{-1}}^{1}
\end{aligned}
$$

Using Proposition 18, we can replace $\boldsymbol{\Lambda}$ by $\boldsymbol{U}^\top\boldsymbol{\Sigma_\theta U}$.

$$
\begin{aligned}
=& |\boldsymbol{U}^\top\boldsymbol{\Sigma_\theta U}|^{-1} \cdot |\left(\boldsymbol{X}^\top\boldsymbol{\Sigma}_\theta^{-1}\boldsymbol{X}\right)^{-1} \overbrace{\boldsymbol{X}^\top\boldsymbol{\Sigma}_\theta^{-1}\boldsymbol{X}\left(\boldsymbol{X}^\top\boldsymbol{\Sigma}_\theta^{-1}\boldsymbol{X}\right)^{-1}}^{\boldsymbol{I}}|^{-1} \cdot |\boldsymbol{X}^\top\boldsymbol{\Sigma}_\theta^{-1}\boldsymbol{X}|^{-1} \\
=& |\boldsymbol{U}^\top\boldsymbol{\Sigma_\theta U}|^{-1} \cdot |\left(\boldsymbol{X}^\top\boldsymbol{\Sigma}_\theta^{-1}\boldsymbol{X}\right)^{-1} \boldsymbol{X}^\top\boldsymbol{\Sigma}_\theta^{-1} \overbrace{\boldsymbol{\Sigma_\theta\Sigma}_\theta^{-1}}^{\boldsymbol{I}} \boldsymbol{X}\left(\boldsymbol{X}^\top\boldsymbol{\Sigma}_\theta^{-1}\boldsymbol{X}\right)^{-1}|^{-1} \cdot |\boldsymbol{X}^\top\boldsymbol{\Sigma}_\theta^{-1}\boldsymbol{X}|^{-1} \\
=& \left|\begin{bmatrix} \boldsymbol{U}^\top\boldsymbol{\Sigma_\theta U} & \boldsymbol{0} \\ \boldsymbol{0} & \underbrace{\left(\boldsymbol{X}^\top\boldsymbol{\Sigma}_\theta^{-1}\boldsymbol{X}\right)^{-1}\boldsymbol{X}^\top\boldsymbol{\Sigma}_\theta^{-1}}_{\boldsymbol{Q}} \boldsymbol{\Sigma_\theta} \underbrace{\boldsymbol{\Sigma}_\theta^{-1}\boldsymbol{X}\left(\boldsymbol{X}^\top\boldsymbol{\Sigma}_\theta^{-1}\boldsymbol{X}\right)^{-1}}_{\boldsymbol{Q}^\top} \end{bmatrix}\right|^{-1} \cdot |\boldsymbol{X}^\top\boldsymbol{\Sigma}_\theta^{-1}\boldsymbol{X}|^{-1}
\end{aligned}
$$

To shorten notation we define the matrix $\boldsymbol{Q} = \left(\boldsymbol{X}^\top\boldsymbol{\Sigma}_\theta^{-1}\boldsymbol{X}\right)^{-1}\boldsymbol{X}^\top\boldsymbol{\Sigma}_\theta^{-1}$.

$$
= \left|\begin{bmatrix} \boldsymbol{U}^\top\boldsymbol{\Sigma_\theta U} & \overbrace{\boldsymbol{U}^\top\boldsymbol{\Sigma_\theta Q}^\top}^{\boldsymbol{0}} \\ \underbrace{\boldsymbol{Q\Sigma_\theta U}}_{\boldsymbol{0}} & \boldsymbol{Q\Sigma_\theta Q}^\top \end{bmatrix}\right|^{-1} \cdot |\boldsymbol{X}^\top\boldsymbol{\Sigma}_\theta^{-1}\boldsymbol{X}|^{-1}
$$

As $\boldsymbol{X}^\top\boldsymbol{U} = \boldsymbol{0}$ it follows that also $\boldsymbol{Q\Sigma_\theta U} = \boldsymbol{0}$.

$$
= \left|\begin{bmatrix} \boldsymbol{U}^\top \\ \boldsymbol{Q} \end{bmatrix} \boldsymbol{\Sigma_\theta} \begin{bmatrix} \boldsymbol{U}^\top \\ \boldsymbol{Q} \end{bmatrix}^\top\right|^{-1} \cdot |\boldsymbol{X}^\top\boldsymbol{\Sigma}_\theta^{-1}\boldsymbol{X}|^{-1}
$$

Using $|\boldsymbol{AB}| = |\boldsymbol{A}| \cdot |\boldsymbol{B}|$ for full rank matrices $\boldsymbol{A}$ and $\boldsymbol{B}$, we get

$$
\begin{aligned}
=& |\boldsymbol{\Sigma_\theta}|^{-1} \cdot \left|\begin{bmatrix} \boldsymbol{U}^\top \\ \boldsymbol{Q} \end{bmatrix} \begin{bmatrix} \boldsymbol{U}^\top \\ \boldsymbol{Q} \end{bmatrix}^\top\right|^{-1} \cdot |\boldsymbol{X}^\top\boldsymbol{\Sigma}_\theta^{-1}\boldsymbol{X}|^{-1} \\
=& |\boldsymbol{\Sigma_\theta}|^{-1} \cdot \left|\begin{bmatrix} \boldsymbol{U}^\top\boldsymbol{U} & \boldsymbol{U}^\top\boldsymbol{Q}^\top \\ \boldsymbol{QU} & \boldsymbol{QQ}^\top \end{bmatrix}\right|^{-1} \cdot |\boldsymbol{X}^\top\boldsymbol{\Sigma}_\theta^{-1}\boldsymbol{X}|^{-1}
\end{aligned}
$$

Using the well-known formula for the determinant of a block-matrix, we get

$$
\begin{aligned}
=& |\boldsymbol{\Sigma_\theta}|^{-1} \cdot |\underbrace{\boldsymbol{U}^\top\boldsymbol{U}}_{\boldsymbol{I}}|^{-1} \cdot |\boldsymbol{QQ}^\top - \boldsymbol{Q}\underbrace{\boldsymbol{U}\left(\boldsymbol{U}^\top\boldsymbol{U}\right)^{-1}\boldsymbol{U}^\top}_{\boldsymbol{S}}\boldsymbol{Q}^\top|^{-1} \cdot |\boldsymbol{X}^\top\boldsymbol{\Sigma}_\theta^{-1}\boldsymbol{X}|^{-1} \\
=& |\boldsymbol{\Sigma_\theta}|^{-1} \cdot |\underbrace{\boldsymbol{QQ}^\top - \boldsymbol{QQ}^\top}_{\boldsymbol{0}} + \underbrace{\boldsymbol{QX}}_{\boldsymbol{I}}\left(\boldsymbol{X}^\top\boldsymbol{X}\right)^{-1}\underbrace{\boldsymbol{X}^\top\boldsymbol{Q}^\top}_{\boldsymbol{I}}|^{-1} \cdot |\boldsymbol{X}^\top\boldsymbol{\Sigma}_\theta^{-1}\boldsymbol{X}|^{-1} \\
=& |\boldsymbol{\Sigma_\theta}|^{-1} \cdot |\boldsymbol{X}^\top\boldsymbol{X}| \cdot |\boldsymbol{X}^\top\boldsymbol{\Sigma}_\theta^{-1}\boldsymbol{X}|^{-1}.
\end{aligned}
$$

□

7.6 Matrix square root of $\boldsymbol{P}_{\boldsymbol{\theta}}$

Let $\boldsymbol{U}\boldsymbol{\Lambda}^{1/2}\boldsymbol{V}^{\top}$ be the economy singular value decomposition of $\boldsymbol{P}_{\boldsymbol{\theta}}^{1/2}$, where the N-by-$(N-D)$ matrix $\boldsymbol{U}$ is the matrix of left singular vectors of $\boldsymbol{P}_{\boldsymbol{\theta}}^{1/2}$ (and eigenvectors of $\boldsymbol{P}_{\boldsymbol{\theta}}$), $\boldsymbol{\Lambda}^{-1/2}$ is the $(N-D)$-by-$(N-D)$ matrix holding the non-zero singular values of $\boldsymbol{P}_{\boldsymbol{\theta}}^{1/2}$ on the diagonal (and a diagonal matrix square root of the matrix $\boldsymbol{\Lambda}^{-1}$ of non-zero eigenvalues of $\boldsymbol{P}_{\boldsymbol{\theta}}$), and $\boldsymbol{V}$ is the matrix of right singular vectors of $\boldsymbol{P}_{\boldsymbol{\theta}}^{1/2}$. Also note that $\boldsymbol{S} = \boldsymbol{U}\boldsymbol{U}^{\top}$, as shown in Lemma 17, that $\boldsymbol{S}\boldsymbol{X} = \boldsymbol{0}$, as shown in Proposition 4, and that $\boldsymbol{S}\boldsymbol{\Sigma}_{\boldsymbol{\theta}}\boldsymbol{S} = \boldsymbol{P}_{\boldsymbol{\theta}}^{\dagger}$, as shown in Lemma 12.

Proposition 20.

$$\boldsymbol{y} \sim \mathcal{N}(\boldsymbol{X}\boldsymbol{\beta}; \boldsymbol{\Sigma}_{\boldsymbol{\theta}}) \;\Rightarrow\; \boldsymbol{P}_{\boldsymbol{\theta}}^{\top/2}\boldsymbol{y} \sim \mathcal{N}(\boldsymbol{0}; \boldsymbol{I}).$$

Proof. An affine transformation of a normally distributed variable is normally distributed. From Proposition 21 it follows that that the expectation of the transformed variable is zero. From Proposition 22 it follows that the covariance matrix of the transformed variable is $\boldsymbol{I}_{N-D}$, completing the proof. □

Proposition 21.

$$\boldsymbol{P}_{\boldsymbol{\theta}}^{\top/2}\boldsymbol{X}\boldsymbol{\beta} = \boldsymbol{0}.$$

Proof.

$$\begin{aligned}
\boldsymbol{P}_{\boldsymbol{\theta}}^{\top/2}\boldsymbol{X}\boldsymbol{\beta} &= \boldsymbol{V}\boldsymbol{\Lambda}^{-1/2}\boldsymbol{U}^{\top}\boldsymbol{X}\boldsymbol{\beta} \\
&= \boldsymbol{V}\boldsymbol{\Lambda}^{-1/2}\overbrace{\boldsymbol{U}^{\top}\boldsymbol{U}}^{\boldsymbol{I}_{N-D}}\boldsymbol{U}^{\top}\boldsymbol{X}\boldsymbol{\beta} \\
&= \boldsymbol{V}\boldsymbol{\Lambda}^{-1/2}\boldsymbol{U}^{\top}\underbrace{\boldsymbol{S}\boldsymbol{X}}_{\boldsymbol{0}}\boldsymbol{\beta} \\
&= \boldsymbol{0}.
\end{aligned}$$

□

Proposition 22.

$$\boldsymbol{P}_{\boldsymbol{\theta}}^{\top/2}\boldsymbol{\Sigma}_{\boldsymbol{\theta}}\boldsymbol{P}_{\boldsymbol{\theta}}^{1/2} = \boldsymbol{I}_{N-D}.$$

Proof.

$$\begin{aligned}
\boldsymbol{P}_{\boldsymbol{\theta}}^{\top/2}\boldsymbol{\Sigma}_{\boldsymbol{\theta}}\boldsymbol{P}_{\boldsymbol{\theta}}^{\top/2} &= \boldsymbol{V}\boldsymbol{\Lambda}^{-1/2}\boldsymbol{U}^{\top}\boldsymbol{\Sigma}_{\boldsymbol{\theta}}\boldsymbol{U}\boldsymbol{\Lambda}^{-1/2}\boldsymbol{V}^{\top} \\
&= \boldsymbol{V}\boldsymbol{\Lambda}^{-1/2}\overbrace{\boldsymbol{U}^{\top}\boldsymbol{U}}^{\boldsymbol{I}_{N-D}}\boldsymbol{U}^{\top}\boldsymbol{\Sigma}_{\boldsymbol{\theta}}\boldsymbol{U}\overbrace{\boldsymbol{U}^{\top}\boldsymbol{U}}^{\boldsymbol{I}_{N-D}}\boldsymbol{\Lambda}^{-1/2}\boldsymbol{V}^{\top} \\
&= \boldsymbol{V}\boldsymbol{\Lambda}^{-1/2}\boldsymbol{U}^{\top}\underbrace{\boldsymbol{S}\boldsymbol{\Sigma}_{\boldsymbol{\theta}}\boldsymbol{S}}_{\boldsymbol{P}_{\boldsymbol{\theta}}^{\dagger}}\boldsymbol{U}\boldsymbol{\Lambda}^{-1/2}\boldsymbol{V}^{\top} \\
&= \boldsymbol{V}\boldsymbol{\Lambda}^{-1/2}\underbrace{\boldsymbol{U}^{\top}\boldsymbol{U}}_{\boldsymbol{I}_{N-D}}\boldsymbol{\Lambda}\underbrace{\boldsymbol{U}^{\top}\boldsymbol{U}}_{\boldsymbol{I}_{N-D}}\boldsymbol{\Lambda}^{-1/2}\boldsymbol{V}^{\top} \\
&= \boldsymbol{V}\underbrace{\boldsymbol{\Lambda}^{-1/2}\boldsymbol{\Lambda}\boldsymbol{\Lambda}^{-1/2}}_{\boldsymbol{I}_{N-D}}\boldsymbol{V}^{\top} \\
&= \boldsymbol{I}_{N-D}.
\end{aligned}$$

□

References

[1] Christoph Lippert. *Linear mixed models for genome-wide association studies.* PhD thesis, Eberhard Karls Universität, Tübingen, 2013.

[2] R. B. Davies. Algorithm as 155: The distribution of a linear combination of χ 2 random variables. *Applied Statistics*, pages 323–333, 1980.

[3] D. A. Harville. Bayesian inference for variance components using only error contrasts. *Biometrika*, 61(2):383–385, 1974.

[4] C. Lippert, J. Listgarten, Y. Liu, C. M. Kadie, R. I. Davidson, and D. Heckerman. FaST linear mixed models for genome-wide association studies. *Nature Methods*, 8(10):833–835, 2011.

[5] J. Listgarten, C. Lippert, C. M. Kadie, R. I. Davidson, E. Eskin, and D. Heckerman. Improved linear mixed models for genome-wide association studies. *Nature Methods*, 9(6):525–526, 2012.

[6] J. Listgarten, C. Lippert, E. Y. Kang, X. Jing, C. M. Kadie, and D. Heckerman. A powerful and efficient set test for genetic markers that handles confounding. *Bioinformatics*, 2013.

[7] H. D. Patterson and R. Thompson. Recovery of inter-block information when block sizes are unequal. *Biometrika*, 58(3):545–554, 1971.
